# A Multi‐Task Self‐Supervised Strategy for Predicting Molecular Properties and FGFR1 Inhibitors

**DOI:** 10.1002/advs.202412987

**Published:** 2025-02-08

**Authors:** Xin Yang, Yang Wang, Ye Lin, Mingxuan Zhang, Ou Liu, Jianwei Shuai, Qi Zhao

**Affiliations:** ^1^ School of Computer Science and Software Engineering University of Science and Technology Liaoning Anshan Liaoning 114051 P. R. China; ^2^ Wenzhou Key Laboratory of Biomedical Imaging Center of Biomedical Physics Wenzhou Institute University of Chinese Academy of Sciences Wenzhou Zhejiang 325001 P. R. China; ^3^ College of Computer Science and Technology Jilin University Changchun Jilin 130012 P. R. China; ^4^ School of Electronic and Information Engineering University of Science and Technology Liaoning Anshan Liaoning 114051 P. R. China; ^5^ Oujiang Laboratory (Zhejiang Lab for Regenerative Medicine, Vision, and Brain Health) Wenzhou Institute University of Chinese Academy of Sciences Wenzhou Zhejiang 325001 P. R. China

**Keywords:** FGFR1, graph neural networks, molecular properties, multi‐task strategy, pretraining framework

## Abstract

Studying the molecular properties of drugs and their interactions with human targets aids in better understanding the clinical performance of drugs and guides drug development. In computer‐aided drug discovery, it is crucial to utilize effective molecular feature representations for predicting molecular properties and designing ligands with high binding affinity to targets. However, designing an effective multi‐task and self‐supervised strategy remains a significant challenge for the pretraining framework. In this study, a multi‐task self‐supervised deep learning framework is proposed, MTSSMol, which utilizes ≈10 million unlabeled drug‐like molecules for pretraining to identify potential inhibitors of fibroblast growth factor receptor 1 (FGFR1). During the pretraining of MTSSMol, molecular representations are learned through a graph neural networks (GNNs) encoder. A multi‐task self‐supervised pretraining strategy is proposed to fully capture the structural and chemical knowledge of molecules. Extensive computational tests on 27 datasets demonstrate that MTSSMol exhibits exceptional performance in predicting molecular properties across different domains. Moreover, MTSSMol's capability is validated to identify potential inhibitors of FGFR1 through molecular docking using RoseTTAFold All‐Atom (RFAA) and molecular dynamics simulations. Overall, MTSSMol provides an effective algorithmic framework for enhancing molecular representation learning and identifying potential drug candidates, offering a valuable tool to accelerate drug discovery processes. All of the codes are freely available online at https:// github.com/zhaoqi106/MTSSMol.

## Graphical abstract

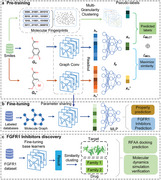

## 1 Introduction

Drug discovery and development are complex and challenging tasks, even with the latest advancements in biomedical research and technology. This complexity arises from the multiple stages involved, each presenting unique challenges and difficulties.^[^ ^1^, ^2^ ^]^ The drug discovery phase requires extensive basic scientific research, including understanding disease mechanisms, identifying potential drug targets, and screening and optimizing compounds.^[^ ^3^, ^4^ ^]^ Traditional experimental methods for evaluating the impact of each compound on all possible protein targets require significant time and resources.^[^ ^5^ ^]^ To overcome these limitations, researchers often use computational methods to predict the potential interactions between compounds and protein targets.^[^ ^6^, ^7^ ^]^ These computational methods, based on protein structure and compound structure, simulate and predict interactions, helping researchers screen for compounds most likely to interact with specific targets, thus reducing the number and cost of experiments.^[^ ^8^ ^]^

In recent years, the rise of advanced artificial intelligence technologies has brought numerous opportunities and breakthroughs to drug design and target identification, demonstrating significant efficiency and cost‐effectiveness.^[^ ^9^, ^10^, ^11^ ^]^ Artificial intelligence can handle large‐scale bioinformatics data, including single‐cell multi‐omics data analysis,^[^ ^12^, ^13^, ^14^, ^15^ ^]^ computational toxicology,^[^ ^16^, ^17^, ^18^ ^]^ miRNA‐lncRNA interactions prediction,^[^ ^19^, ^20^ ^]^ metabolite‐disease associations prediction,^[^ ^21^, ^22^ ^]^ and remote health monitoring.^[^ ^23^, ^24^, ^25^ ^]^ Through machine learning and deep learning techniques, patterns and regularities can be discovered from these data, enabling the prediction of interactions between drugs and targets, thus accelerating the process of drug design.^[^ ^26^, ^27^ ^]^ Learning molecular representations from chemical structures is a fundamental challenge in utilizing artificial intelligence methods for predicting molecular properties.^[^ ^28^ ^]^

However, traditional methods for molecular representation, such as those based on manually crafted features, physical‐chemical descriptors, and pharmacophore‐based features, typically rely on the expertise of domain specialists to extract molecular features. While these methods may perform well in certain cases, they also have limitations. They require a deep understanding of chemical structures and properties to manually select and extract features, thereby limiting their applicability and generalization, especially for complex molecular structures and unknown compounds.^[^ ^29^, ^30^, ^31^ ^]^ Self‐supervised learning (SSL) methods have emerged as a powerful approach to molecular property prediction, leading to significant advancements in molecular representation learning.^[^ ^32^, ^33^ ^]^ SSL methods have been applied to pre‐train GNNs to improve molecular representation learning. By learning from large‐scale unlabeled compound data, these methods can acquire richer and more effective molecular representations, thereby enhancing the performance of downstream molecular property prediction tasks.^[^ ^34^, ^35^, ^36^ ^]^

Currently, SSL methods such as GROVER^[^ ^37^ ^]^ and MolCLR^[^ ^38^ ^]^ modify molecular graphs and then predict the modified parts or align the modified graphs with corresponding original graphs in latent space through contrastive learning. However, contrastive learning objectives may be highly sensitive to minor modifications of graphs. Even slight structural changes can make it difficult for the contrastive learning model to align the modified graph with the original graph, thereby reducing the robustness and generalization ability of the model. Additionally, contrastive learning objectives may lead to overfitting specific representations of the original graph, neglecting broader semantic information in molecular structures. This could result in poor performance of the model on new, unseen molecules. Taking these factors into account, while contrastive learning objectives can assist the model in learning molecular representations to some extent, there are still challenges and limitations. Moreover, most existing SSL‐based approaches predominantly rely on a single SSL task to train GNNs for drug discovery, which introduces a bias toward that task. Although Wang et al. proposed a multi‐task SSL‐based strategy for biomedical network drug discovery (MSSL2drug),^[^ ^39^ ^]^ their method primarily emphasizes the relationships among drug molecules, proteins, and diseases, while overlooking the structural characteristics inherent to drug molecules themselves.

In this study, we propose a multi‐task self‐supervised deep learning framework named MTSSMol, specifically designed for predicting molecular properties and designing high‐affinity ligands. MTSSMol integrates two pre‐training strategies and unsupervised learning, utilizing large‐scale unlabeled data for training to learn and optimize molecular representations. Compared to existing methods, MTSSMol excels in effectively handling various drug discovery tasks and demonstrates exceptional performance in predicting the molecular properties of targets such as FGFR1. Our research demonstrates that MTSSMol not only achieves significant performance in theoretical predictions but also undergoes validation through RFAA and molecular dynamics simulations, thus providing a robust computational framework to accelerate drug discovery.

## 2 Materials and Methods

### 2.1 Model Framework

**Figure 1:**
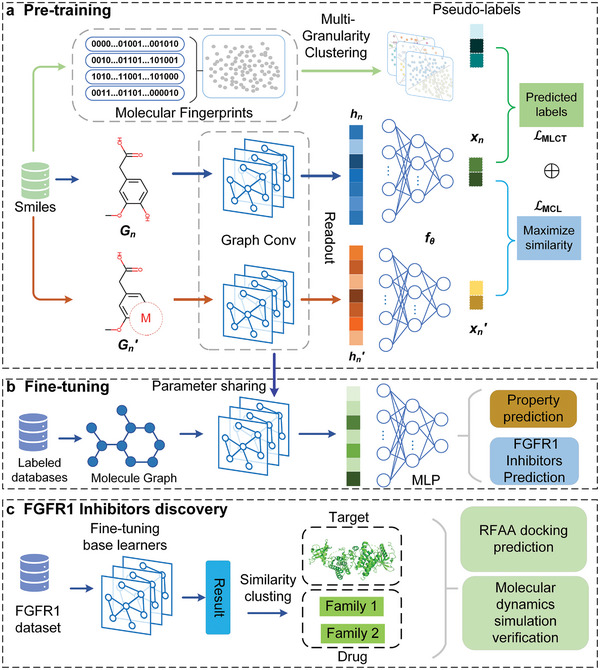
a) The overview of MTSSMol during pretraining. Initially, data augmentation techniques are applied to the raw input data to create various enhanced versions. These images are then fed into GNNs to extract latent features. Finally, the augmented data is utilized for two learning tasks. b) Transfer learning for downstream molecular property prediction in the feature extraction setup. c) FGFR1 inhibitor discovery process.

We develop a pre‐training deep learning framework called MTSSMol to accurately predict molecular properties. To accurately capture the structural information of molecules, we design two pre‐training tasks aimed at learning biologically relevant features: 1) we create GNNs molecular encoder to extract latent features from ≈10 million molecules; 2) by employing two pre‐training strategies that consider chemical knowledge and structural information in molecular graphs, we optimize the latent representations of molecular encoder (**Figure** **1**a); 3) the pretrained GNNs module of MTSSMol is fine‐tuned on target molecular property datasets using supervised learning, refining the entire model for molecular property prediction (Figure 1b) and thus further enhancing its performance;^[^ ^40^, ^41^ ^]^ 4) as shown in Figure 1c, we fine‐tune the pretrained model using a known FGFR1 molecular dataset to predict potential inhibitors for this target. The predicted results are then filtered and followed by similarity clustering, resulting in two distinct molecular families. Subsequently, we employ RFAA to predict the docking results of these two families with the target. Finally, molecular dynamics simulations are conducted to validate the docking results.

### 2.2 Molecule Graph Augmentation

#### 2.2.1 Assign Labels to Molecular Diagrams

The main purpose of using multi‐granularity clustering and pseudo‐labels is to enhance the model's expressiveness and generalization by capturing different levels of similarity in chemical structures and reducing dependence on labeled data. Multi‐granularity clustering provides richer structural information, while pseudo‐labels allow the use of extensive unlabeled data, thereby improving the model's robustness and predictive performance. As shown in Figure 1a, in simple terms, multi‐granularity clustering is initially utilized to assign multiple different granularity clusters to each chemical structure fingerprint. Subsequently, each cluster is assigned as a pseudo‐label to the corresponding molecule, resulting in each molecule having multiple pseudo‐labels of varying granularities. Specifically, we utilize MACCS fingerprints to represent molecules, which consist of a 166‐bit sequence of 0 and 1. These molecular fingerprints form the basis for clustering: molecules with similar fingerprints are more likely to belong to the same cluster. We apply K‐means clustering with different values of K (100, 1000, and 10000), resulting in clusters at varying levels of granularity, from coarse to fine. Based on the clustering outcomes, each molecular graph is assigned three distinct pseudo‐labels.

#### 2.2.2 Graph Mask

The masking process starts with randomly selected initial atoms in the graph *G_n_*. Masking then extends to neighboring atoms and their subsequent neighbors until the proportion of masked atoms reaches a predetermined ratio of the total atom count. Subsequently, the bonds between the masked atoms are removed, forming a subgraph of the original molecule induced by the masked atoms and deleted bonds. As shown in Figure 1a, the masked graph is denoted as *G_n_* ***^’^***.

### 2.3 Graph Neural Networks

Specifically, we abstract the molecule represented by SMILES into a molecular graph *G = (V, E)*, where atoms are represented as nodes *V* and bonds are represented as edges *E* (see Table S1, Supporting Information). The core idea of GNNs is to propagate and aggregate information through the connections between nodes, thereby updating the representation of each node. Through multiple rounds of message passing and aggregation operations, GNNs can capture complex local and global features in graph data, gradually enhancing the representation capability of nodes. AGGREGATE(·) and COMBINE(·) are two fundamental operations of GNNs, where AGGREGATE(·) retrieves message information from the neighbors of node *v*, and COMBINE(·) integrates the information from all neighbors of node *v*. The message‐passing process in the *k*‐th layer of GNNs is shown as follows:

(1) $$a_{v}^{\left(k\right)}=AGGREGATE^{\left(k\right)}\left(\left\{h_{u}^{\left(k-1\right)}:u\in N\left(v\right)\right\}\right)$$

(2) $$h_{v}^{\left(k\right)}=COMBINE^{\left(k\right)}\left(h_{v}^{\left(k-1\right)},a_{v}^{\left(k\right)}\right)$$

where $a_{v}^{\left(k\right)}$ represents the feature formed by aggregating the features of neighboring nodes for node *v*, *N(v)* is the set of neighboring nodes for node *v*, and $h_{v}^{\left(k\right)}$ is the feature of node *v* at the *k*‐th layer. In order to further extract the graph‐level feature *h_G_*, the readout operation integrates all node features in graph *G*, as shown below:

(3) $$h_{G}=READOUT\left(\left\{h_{v}^{\left(k\right)}:v\in G\right\}\right)$$

In our model, we build a GNNs encoder based on a graph isomorphism network (GIN). In GIN, the node aggregation operation typically involves a weighted summation of neighbor node features combined with the node's own features. Through multiple rounds of node aggregation operations, GIN gradually updates the node representations, capturing complex relationships and patterns in the graph data. Furthermore, we utilize mean pooling for the readout operation to obtain the representation of the entire graph.

### 2.4 Strategies for Pretraining MTSSMol

The purpose of pretraining is to enable models to acquire extensive knowledge and language understanding across various tasks through learning from large‐scale datasets. This type of pretraining helps models learn language grammar, semantic relationships, and rich knowledge, thereby enhancing their language comprehension and generation capabilities. Through pre‐training, models can learn rich contextual information to better understand and generate natural language. In this work, we define two effective and task‐related proxy tasks for pre‐training models.

#### 2.4.1 Multi‐Label Classification Task

Based on semantic consistency, we propose a multi‐label classification task for discovering semantic consistency by predicting molecular chemical structures. Specifically, first, we assign three pseudo‐labels to the molecular graph using data augmentation methods. Then, we use the molecular graph features as output and employ GNNs molecular encoder to extract the latent features of the molecular graph, which are then used to predict pseudo‐labels via a structural classifier. This classifier comprises three parallel fully connected layers, each corresponding to a different label classification. The number of neurons in these layers is set to 100, 1000, and 10000, respectively, based on different clustering requirements. Finally, the molecular graph features of each sample are represented as *x_n_*, and their three pseudo‐labels are represented as $y_{n}^{100}\in {\left\{0,1,\text{…},99\right\}}^{100}$, $y_{n}^{1000}\in {\left\{0,1,\text{…},999\right\}}^{1000}$ and $y_{n}^{10000}\in {\left\{0,1,\text{…},9999\right\}}^{10000}$. The loss function is defined as:

(4) $$\begin{array}{ccc} L_{MLCT} & = & \underset{\theta ,W}{\arg\min\frac{1}{N}}\sum_{n=1}^{N}\left[\ell_{1}\left(W_{100}f_{\theta }\left(x_{n}\right),y_{n}^{100}\right)\right.\\ & & \left.+\ell_{2}\left(W_{1000}f_{\theta }\left(x_{n}\right),y_{n}^{1000}\right)+\ell_{3}\left(W_{10000}f_{\theta }\left(x_{n}\right),y_{n}^{10000}\right)\right] \end{array}$$

where *ℓ_1_, ℓ*_2_, *and ℓ _3_* represent the losses for multi‐label classification, using the CrossEntropyLoss function for calculating the cross‐entropy loss. The parameters of three fully connected classification layers in the structural classifier are denoted as *W_100_, W_1000,_* and *W_10000_*, corresponding to layers with 100, 1000, and 10000 neurons, respectively. *f_θ_* represents the mapping function of the molecular encoder.

#### 2.4.2 Mask‐Based Contrastive Learning

Traditional contrastive learning learns the underlying structure and relationships of data by comparing the similarity between different samples, capturing the intrinsic characteristics of the data.^[^ ^38^, ^42^ ^]^ However, this process is computationally intensive. Additionally, to fully leverage the feature extraction capabilities of pre‐trained models, contrastive learning has high requirements for selecting feature pairs, which results in the need for significant computational resources.^[^ ^43^ ^]^ To address the issues mentioned above, we introduce a simple graph comparison learning method, namely masked graph contrastive learning. This approach is a straightforward and effective way to compare graphs, enabling conservation of computational resources and extraction of more detailed chemical structural information from molecular graphs. By using masks, the focus is placed on the shared parts when comparing two graphs, thereby enhancing efficiency and reducing computational burden. This method plays a crucial role in fields such as molecular structure alignment and similarity analysis, aiding in a deeper understanding of the relationships between molecules. In addition, introducing the cost function ℒ*_MC_*_L_ ensures the consistency of the potential features extracted from the molecular graphs before and after masking. Its form is shown below:

(5)

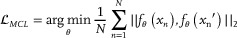

where, *x*_n_ and *x*_n_′ represent the original molecular graph and the molecular graph after masking, respectively. ||*f*_θ_(*x_n_*),*f*_θ_(*x_n_* ′)||_2_ marks the calculation of Euclidean distance between *x_n_* and  *x_n_* ′.

#### 2.4.3 Training Details

Here, molecular graphs are obtained through data preprocessing and forwarded to GNNs to extract latent features *h_n_*. Each atom on the molecular graph is embedded based on its atomic number and chirality type, while each bond is embedded based on its type and direction. We implement a 5‐layer graph convolution with ReLU activation as the backbone of GNNs to ensure compatibility between aggregation and edge features. Average pooling is applied on each graph as the readout operation to extract a 512‐D molecular representation. An MLP with one hidden layer maps the representation to a 256‐D latent space. Subsequently, utilizing these latent features, the total cost function ℒ*_all_* is calculated through two auxiliary tasks:

(6) $$L_{all}=L_{MLCT}+L_{MCL}$$

Finally, we utilize ℒ*_all_* for backpropagation to update GNNs. Specifically, we use Adam optimization to minimize the cost function.^[^ ^44^ ^]^ The pre‐training of MTSSMol took ≈40 h on two Nvidia 4090 GPUs. For more detailed hyperparameter settings, please refer to Table S2 (Supporting Information).

### 2.5 Fine‐Tuning

To evaluate MTSSMol, we designed four downstream prediction tasks related to molecular representation learning: molecular property prediction, therapeutics data commons (TDC) prediction, drug metabolism prediction, and anti‐FGFR1 target inhibitors prediction. In the first task of molecular property prediction, we use 11 benchmark datasets from MoleculeNet,^[^ ^6^ ^]^ including 6 classification datasets and 5 regression datasets (see Table S3, Supporting Information). For the second prediction task, the data comes from the TDC benchmark dataset, which provides 9 datasets measuring the absorption, distribution, and toxicity of molecules.^[^ ^45^ ^]^ In drug metabolism prediction, we utilize the PubChem dataset from Cheng et al.^[^ ^46^ ^]^ which includes 5 human CYP subtypes. In FGFR1 target prediction, we fine‐tune the model using experimental data from Li et al.^[^ ^47^ ^]^ and then collect FDA‐approved drugs from DrugBank (referred to as the FDA dataset).

### 2.6 FGFR1‐Ligand Complex: Structure Prediction to Binding Energy

We predict FGFR1 and ligand complex structure by the latest and highly accurate deep learning‐based protein structure prediction tool RFAA and AlphaFold2‐multimer.^[^ ^48^, ^49^, ^50^, ^51^ ^]^ This structure is then subjected to all‐atom molecular dynamics (MD) simulations using GROMACS to assess its stability and dynamic behavior under physiological conditions. Binding free energy is estimated using the gmx_MMPBSA tool, with key residue contributions analyzed through energy decomposition. Detailed descriptions of MD simulation setup, system parameters, and binding energy calculation are provided in Note S1.8 (Supporting Information).

## 3 Results

### 3.1 Benchmark Evaluation of MTSSMol

To demonstrate the effectiveness of MTSSMol, we benchmark its performance on multiple challenging classification and regression tasks from MoleculeNet. These datasets include: blood‐brain barrier penetration (BBBP); β‐secretase (BACE, a key target for Alzheimer's disease); human immunodeficiency virus (HIV); side effect resource (SIDER); molecular toxicity (Tox21) and clinical trial toxicity (ClinTox) for toxicology studies; solubility: Free solvation (FreeSolv) and estimated solubility (ESOL) and lipophilicity.

We first employ the multi‐task pre‐training strategy to build predictive models using GCN,^[^ ^38^ ^]^ GAT^[^ ^39^ ^]^ and GIN architectures. Evaluation results indicate that GIN consistently achieves superior performance across multiple classification and regression tasks. Table S4 (Supporting Information) summarizes the performance of these models on six classification tasks evaluated using AUC scores. GIN exhibits higher predictive accuracy compared to GCN and GAT across all datasets. Specifically, on BACE, GIN achieves AUC = 0.915, outperforming GAT (AUC = 0.891) and GCN (AUC = 0.885) by 2.7% and 3.4%, respectively. For BBBP, GIN achieves AUC = 0.930, improving upon GAT (AUC = 0.885) by 5.1% and GCN (AUC = 0.915) by 1.6%. On ClinTox, GIN records AUC = 0.842, surpassing GAT (AUC = 0.816) by 3.2% and GCN (AUC = 0.839) by 0.4%. For HIV, GIN achieves AUC = 0.834, outperforming GAT (AUC = 0.771) by 8.2% and GCN (AUC = 0.787) by 6.0%. On SIDER, GIN scores AUC = 0.643, which is slightly better than GAT (AUC = 0.642) by 0.2% and outperforms GCN (AUC = 0.622) by 3.4%. For Tox21, GIN achieves AUC = 0.857, exceeding GAT (AUC = 0.801) by 7.0% and GCN (AUC = 0.828) by 3.5%. GIN also consistently outperforms GAT and GCN across all regression tasks (Table S5, Supporting Information). For RMSE evaluations, GIN achieves the lowest errors on FreeSolv (RMSE = 1.209), ESOL (RMSE = 0.798), and Lipo (RMSE = 0.65), with improvements of 37.2%, 21.4%, and 12.9% over GAT, and 38.4%, 11.9%, and 12.5% over GCN, respectively. Similarly, in MAE evaluations, GIN demonstrates superior performance on QM7 (MAE = 67.4) and QM8 (MAE = 0.0125), surpassing GAT by 15.1% and 2.3%, and GCN by 12.8% and 17.8%, respectively. These results underscore GIN's ability to generate more accurate molecular representations, leading to consistent improvements across diverse regression tasks.

**Figure 2:**
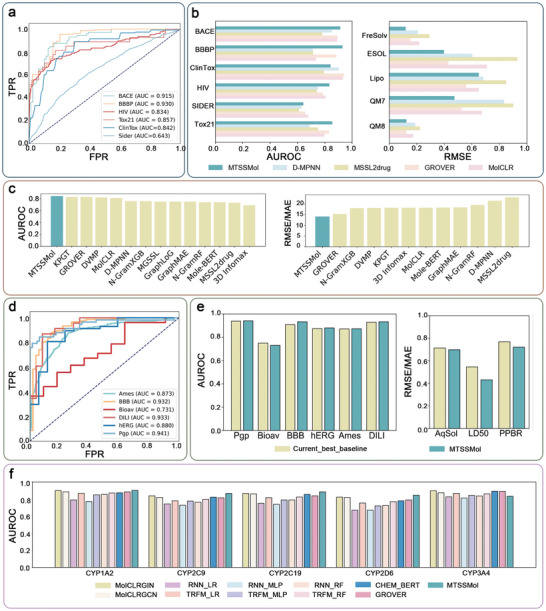
a) AUROC curves of MTSSMol on 6 classification datasets (BBBP, Tox21, HIV, ClinTox, BACE, SIDER). b) AUROC and RMSE/MAE performance are compared with four SOTA models and MTSSMol on six classification datasets and five regression datasets. c) The performance of MTSSMol and the comparative algorithms on 6 classification datasets and 5 regression datasets. d) AUROC curves of MTSSMol on TDC classification datasets (Pgp, Bioav, BBB, hERG, Ames, DILI). e) MTSSMol's predictive performance on the TDC benchmark dataset, as well as the best baseline methods provided by the TDC benchmark leaderboard. Left: AUROC results for binary classification tasks. Right: RMSE/MAE results for regression tasks (AqSol, LD50, PPBR). f) AUROC performance of MTSSMol and comparative algorithms on CYP450 datasets (CYP1A2, CYP2C9, CYP2C19, CYP2D6, CYP3A4).

Consequently, GIN is selected as the baseline model for subsequent experiments and comparisons. In classification tasks, we achieve higher AUC values across all tasks (**Figure** **2**a). Next, we compare the performance of MTSSMol with four state‐of‐the‐art (SOTA) self‐supervised learning methods in the field: D‐MPNN, GROVER, MolCLR, and MSSL2drug. MSSL2drug is a multi‐task self‐supervised model originally designed for predicting interactions among drug molecules, proteins, and diseases.^[^ ^39^ ^]^ To adapt it for molecular property prediction tasks, we modify its input and output settings. These modifications enable us to evaluate the generalizability of MTSSMol by using the adapted MSSL2drug model as a benchmark. On most classification datasets, MTSSMol outperforms the comparison models (Figure 2b). In regression datasets, MTSSMol demonstrates superior performance over all comparison models. Additionally, from Figure 2c, it can be observed that MTSSMol has a higher average AUC than the comparative algorithms across the six classification tasks. Specifically, MTSSMol outperforms the second‐rank method by 2.35% on BACE (AUC = 0.915), 2.4% on BBBP (AUC = 0.930), 2.5% on HIV (AUC = 0.834), and 2.3% on Tox21 (AUC  = 0.857). In regression tasks, MTSSMol also achieves lower RMSE values compared to the comparative algorithms. Specifically, MTSSMol outperforms the second‐rank method by 21.7% on FreSolve (RMSE = 1.209), 2.3% on ESOL (RMSE = 0.798), and 7.2% on QM7 (MAE = 67.4). AUC and confusion matrices are provided in Tables S4, S5, and Figure S1 (Supporting Information). These experimental results indicate that MTSSMol can capture more of the biological information that determines molecular properties from molecular graphs.

Next, we compare the performance of MTSSMol against both machine learning and deep learning methods using the TDC dataset, which provides a benchmarking platform with nine molecular property prediction tasks. These tasks cover key areas such as absorption, distribution, and toxicity, essential for drug discovery and development. The results show that MTSSMol outperforms the current baseline methods on eight out of nine datasets. MTSSMol has higher AUC values on six classification datasets (Figure 2d). Specifically, MTSSMol outperforms the current baseline methods by 0.3%, 2.8%, 0.7%, 0.2%, and 0.4% on Pgp (AUC = 0.941), BBB (AUC = 0.933), hERG (AUC = 0.880), Ames (AUC = 0.873), and DILI (AUC = 0.933), respectively. AUC and confusion matrices are provided in Table S6, and Figure S2 (Supporting Information). MTSSMol outperforms the current baseline methods by 2.3%, 6.2%, and 20.9% on Aqsol (MAE = 0.698), PPBR (MAE = 7.21), and LD50 (MAE = 0.431) regression datasets, respectively. Figure 2e displays the AUC ranking of MTSSMol in each dataset. These findings underscore the robust and versatile molecular representation capabilities of MTSSMol, showcasing its ability to predict a wide range of molecular properties with reliability and generalizability.

We also assess the performance of MTSSMol in the more demanding CYP50 task. In drug discovery, accurately classifying cytochrome P450 inhibitors and non‐inhibitors is vital for predicting significant drug interactions resulting from CYP inhibition and identifying the affected subtypes. This poses a substantial challenge for predictive models. As shown in Figure 2f, MTSSMol demonstrates superior performance compared to the other 11 benchmark algorithms. Furthermore, as illustrated in Figure S4 (Supporting Information), MTSSMol achieves higher AUC values for CYP1A2 (AUC = 0.902), CYP2C9 (AUC = 0.866), CYP2C19 (AUC = 0.885), CYP2D6 (AUC = 0.845), and CYP3A4 (AUC = 0.832), along with superior performance across other performance metrics. Confusion matrices are provided in Figure S3 (Supporting Information). In addition, we also calculate other metrics for CYP50 datasets, and the results demonstrate that MTSSMol exhibits superior performance (Table S7, Supporting Information).

### 3.2 Investigation of MTSSMol Representation

Having demonstrated the superiority of MTSSMol in molecular property prediction, we further investigate the underlying reasons for its exceptional performance. To uncover this, we analyze the latent space generated by the pre‐trained MTSSMol. For this purpose, we utilize a dataset containing 11725 molecules targeting CYP1A2 enzyme and their corresponding bioactivity measurements (i.e., inhibition or non‐inhibition). CYP1A2 enzyme is crucial in drug metabolism.

First, we employ the pre‐trained MTSSMol to generate molecular feature representations for CYP1A2 dataset. We then randomly select 200 molecules from this dataset to serve as a test set, with the remaining molecules used for training. Next, we use k‐nearest neighbors (kNN) classification based on molecular feature representations to predict the activity of the test set molecules. We compare the performance of MTSSMol with two widely used classical fingerprint methods: ECFP and RDKFP.

**Figure 3:**
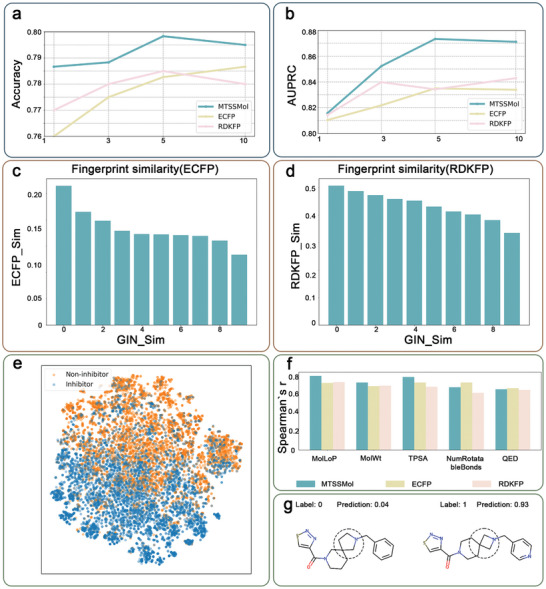
a, b) AUPRC and accuracy of k‐nearest neighbor classification for predicting drug metabolism given different values of k on the CYP1A2 dataset. c, d) Change of ECFP and RDKFP similarities with respect to the distance between MTSSMol representations. e) t‐SNE visualization of molecular representations from CYP1A2 dataset produced by MTSSMol. f) The Spearman's r between five descriptors (i.e., MolLogP, MolWt, TPSA, NumRotatableBonds, QED, and SA) of CYP1A2 dataset and their corresponding closest molecules identified using fingerprints from MTSSMol. g) Activity cliffs identified by SubgraphX.^[^ ^57^ ^]^ Dashed circles highlight the distinguished substructures within the activity cliffs.

**Figure** **3**a,b presents a comparison of MTSSMol with baseline methods, evaluating their performance in terms of accuracy and the area under the precision‐recall curve (AUPRC). Notably, MTSSMol achieves a 3.5% to 4.8% improvement in AUC compared to the baseline methods. These results suggest that the molecular neural fingerprints generated by MTSSMol are more effective in capturing the intricate relationship between molecular structure and biological activity. The latent space constructed by MTSSMol successfully embeds molecules into a continuous vector space, where molecules with similar biological properties exhibit a tendency to cluster. This latent representation offers a robust foundation for downstream tasks such as molecular property prediction and drug design.

**Figure 4:**
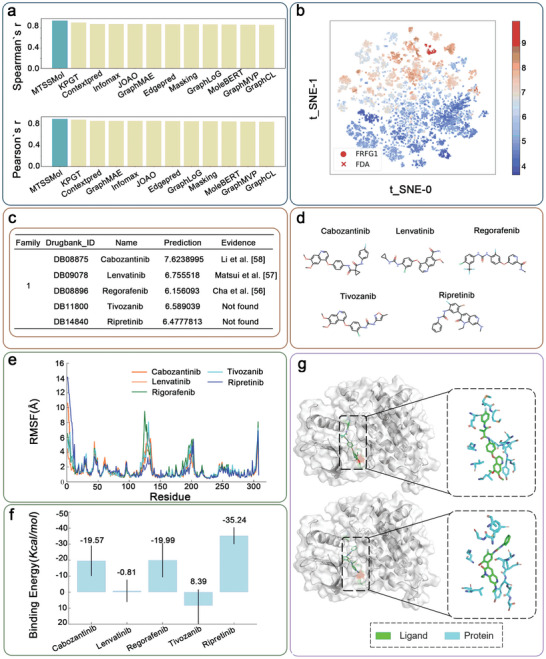
a) The performance of MTSSMol and baseline methods in predicting FGFR1 inhibitors is measured by Pearson's r and Spearman's r, respectively. b) Visualization of molecular representations of molecules from the pIC50 dataset of FGFR1 inhibitors and FDA dataset derived from KPGT. c) Details of 5 molecules in family 1. d) Visualization of 5 molecular structures in family 1. e) RMSF analysis of FGFR1 residues in the presence of different inhibitors. The y‐axis represents RMSF values in Ångstroms (Å), and the x‐axis represents the residue number. f) Binding free energy calculations for each inhibitor. The bar graph shows the average binding energy in Kcal/mol, with error bars representing the standard deviation. Error bars represent standard deviations. g) Visualization of interactions of Cabozantinib and Ripretinib with FGFR1. The protein‐ligand structure (PDB ID: 5A4C) is utilized as a reference for binding pocket identification.

To further evaluate MTSSMol, we compare its learned representations with those of ECFP and RDKFP. Specifically, given a query molecule, we extract its representation using MTSSMol and compute its cosine distance to all reference molecules in a pre‐trained database. The reference molecules are then ranked according to their representation distance and evenly divided into 10 bins based on the ranking percentile, where lower percentiles correspond to higher similarity. Within each bin, 1000 molecules are randomly selected, and their Tanimoto FP similarity to the query is calculated. The similarity distributions using ECFP and RDKFP are shown in **Figure** **4**a,b. ECFP tends to yield lower similarity values compared to RDKFP, as the former encompasses a broader range of features related to molecular activity. However, both ECFP and RDKFP similarities decrease as MTSSMol representation distance increases. Although there are fluctuations with increasing percentile thresholds, the overall trend between MTSSMol representations and chemical fingerprints remains consistent. The distance between MTSSMol representations effectively reflects molecular similarity.

Next, we fine‐tune MTSSMol using the CYP1A2 dataset and employ t‐distributed stochastic neighbor embedding (t‐SNE) to visualize the molecular representations. The fine‐tuned MTSSMol effectively distinguishes CYP1A2 inhibitors from non‐inhibitors, indicating that our model successfully captures the key differences between different types of molecules during the learning process (Figure 3e). To further validate the effectiveness of MTSSMol, we calculate the distance between each pair in the latent space. We then perform correlation analysis on several key properties of these molecule pairs, including molecular LogP (MolLogP), molecular weight (MolWt), topological polar surface area (TPSA), number of rotatable bonds (NumRotatableBonds), quantitative estimate of drug‐likeness (QED), and synthetic accessibility (SA). The results show a high correlation between the molecular representations learned by MTSSMol and these properties, demonstrating that our model effectively captures the relationship between molecular structure and properties. The results in Figure 3f and Figure S5 (Supporting Information) show that MTSSMol performs exceptionally well in predicting molecular properties. By visualizing the latent space, we find that MTSSMol accurately identifies activity cliffs, providing interpretability for the model's predictions (see Figure 3g; Figure S6, Supporting Information). This means we can understand the model's decision‐making process by analyzing the molecular representations in the latent space, thereby better explaining the model's predictive outcomes.

### 3.3 Uncovering Effective Inhibitors for FRFR1 Targets with MTSSMol

FGFR1 is closely associated with various types of cancers and has been extensively studied for anti‐tumor therapy. The availability of high‐quality experimental data on FGFR1 has greatly facilitated the development and validation of artificial intelligence‐based computational models, providing ample data support for assessing the utility and predictive performance of MTSSMol. In this subsection, we conduct evaluation tests, drug repositioning, and docking analyses targeting FGFR1 to validate the effectiveness of MTSSMol in real‐world drug discovery scenarios.

We initially collected 12461 existing FGFR1 molecules from patents and previous experiments. Subsequently, we employ MTSSMol for drug repositioning to identify potential FGFR1 inhibitors. Specifically, we obtained 2718 FDA‐approved drugs from DrugBank (designated as FDA dataset). We then fine‐tune MTSSMol using a dataset of pIC50 values for FGFR1 inhibitors and predict molecules from the FDA dataset. We have comprehensively evaluated MTSSMol's predictive performance on this dataset. Figure 4a presents a detailed comparison of MTSSMol with 11 self‐supervised learning baselines. The results show that MTSSMol significantly outperforms all 11 self‐supervised learning baselines in terms of both Spearman's rank correlation coefficient (Spearman's r) and Pearson correlation coefficient (Pearson's r). Next, we visualize the molecular representations from the FGFR1 inhibitors pIC50 dataset and FDA dataset (Figure 4b) to assess structural similarities and clustering patterns. The visualization highlights distinct clusters of FGFR1 inhibitors, suggesting meaningful groupings based on molecular features captured by MTSSMol. Furthermore, we perform a structural analysis of the top 25 predicted inhibitors identified during drug repurposing. Among them, 15 molecules have been experimentally validated as high‐affinity or effective FGFR1 inhibitors (Table S8, Supporting Information). To further evaluate biological relevance, we examine the functional groups and pharmacophores of these predicted inhibitors and compare them with known FGFR1 inhibitors. Results indicate that the predicted molecules share key structural motifs, such as aromatic scaffolds, hydrogen bond donors/acceptors, and lipophilic regions, which are critical for FGFR1 binding. These findings reinforce MTSSMol's ability to prioritize biologically relevant compounds. Additionally, we cluster these 25 molecules into structural families and identify two main clusters with Tanimoto similarities greater than 0.65 in the FDA database (Table S9, Supporting Information). These clusters exhibit a strong structural resemblance to existing FGFR1 inhibitors, further validating the biological relevance of MTSSMol's predictions.

Docking experiments utilize protein‐ligand structures (PDB ID: 5A4C) as references for binding pocket recognition. Figure 4c,d show detailed information and visualizations of five molecules of family 1, including previously demonstrated FGFR1 inhibitors and potential inhibitors that have not been experimentally demonstrated.^[^ ^52^, ^53^, ^54^ ^]^ Figure 4e shows Root Mean square fluctuation (RMSF) plots, which indicate the flexibility of each residue in the protein when bound to different inhibitors. Higher RMSF values indicate greater flexibility. This plot helps identify regions of the protein that are more dynamic or stabilized by each inhibitor. Notably, residues around positions 50, 150, and 250 show increased fluctuation, suggesting these regions might be critical for inhibitor binding and protein function. The root‐mean‐square deviation (RMSD) plots over time for the protein‐ligand complexes show the overall stability of these complexes during the simulation (Figure S7, Supporting Information). The lower the RMSD value, the better the stability. This plot shows that Cabozantinib and Regorafenib complexes are more stable compared to Tivozanib and Ripretinib, which exhibit higher RMSD values, indicating more significant conformational changes over time. Figure S8 (Supporting Information) presents the RMSD plots for the ligands within the binding pocket over the simulation period. Similar to Figure S7 (Supporting Information), this plot assesses the stability of ligands. The data shows that while most ligands remain relatively stable, Tivozanib exhibits higher fluctuations, indicating potential instability or conformational flexibility within the binding site. The bar graph (Figure 4f) displays the binding energies (in Kcal/mol) for each inhibitor. Ripretinib shows the highest binding energy (−35.24 Kcal mol^−1^), indicating the strongest binding affinity to the target protein. In contrast, Lenvatinib has the lowest binding energy (−0.81 Kcal mol^−1^), suggesting a weaker interaction. These results provide insight into the relative binding strengths of inhibitors, which can inform drug efficacy and design strategies. Figure 4g shows that the Cabozantinib and Ripretinib ligands bind closely to the protein FGR1.

The designed inhibitors from family 1 play a crucial role in modulating the activity of target proteins, often used in therapeutic settings to treat various diseases, including cancer, by blocking specific protein functions. The analysis of these interactions sheds light on the stability, flexibility, and binding affinity of these inhibitors, providing valuable insights into their potential efficacy and mechanisms of action. Together, these analyses help elucidate the mechanistic basis of inhibitor binding and can guide the development of more effective FGFR1 inhibitors.

**Figure 5:**
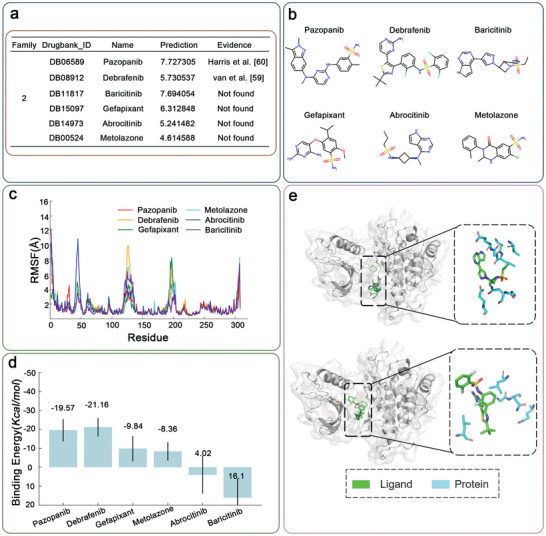
a) Details of 5 molecules in family 2. b) Visualization of 5 molecular structures in family 2. c) RMSF of FGFR1 residues in the presence of different ligands. d) Binding free energy (in Kcal/mol) of the protein‐ligand complexes calculated over the simulation period. The error bars represent the standard deviation of the binding energy value. e) Visualization of interactions of Baricitinib and Debrafenib with FGFR1. The protein‐ligand structure (PDB ID: 5A4C) is utilized as a reference for binding pocket identification.

Next, as a second prototypical family, **Figure** **5**a,b shows the specific information of six molecules in family 2 and their corresponding structure diagrams, two of which have been identified as FGFR1 inhibitors in previous studies.^[^ ^55^, ^56^ ^]^ Figure 5c shows an RMSF plot that measures the flexibility of each residue in the protein over the simulation time for different inhibitors. The plot indicates regions of the protein that are more flexible or more rigid in the presence of each inhibitor. The flexibility varies across different regions of the protein, with certain inhibitors (e.g., Abrocitinib represented in blue) causing higher fluctuations in specific regions. Figure S9 (Supporting Information) shows the RMSD of the protein's backbone atoms over time, indicating the overall stability of the protein structure in the presence of each inhibitor. Inhibitors like Debrafeinib and Pazopanib maintain lower RMSD values, suggesting they stabilize the protein structure more effectively compared to others. Figure S10 (Supporting Information) shows the RMSD of ligands (inhibitors) themselves, indicating how stable the inhibitors are within the binding site over time. Lower RMSD values for the ligands suggest tighter binding and less movement within the binding pocket. Inhibitors such as Debrafeinib and Pazopanib exhibit lower ligand RMSD values, indicating more stable binding interactions compared to others like Metolazone, which shows higher fluctuations. Figure 5d represents the binding energies (in Kcal/mol) of the inhibitors to the protein, with error bars showing the variability. More negative values indicate stronger binding affinities. Debrafeinib and Pazopanib show the most negative binding energies, suggesting they have the highest binding affinities. On the other hand, Metolazone has a positive binding energy, indicating weaker or less favorable binding.

The combination of structural visualization (Figure 5e), flexibility analysis (Figure 5c), overall stability (Figure S9, Supporting Information), ligand stability (Figure S10, Supporting Information), and binding affinity (Figure 5d) provides a comprehensive understanding of how different inhibitors interact with FGFR1. Inhibitors like Debrafeinib and Pazopanib are highlighted as potentially more effective due to their strong binding affinities, stability, and ability to maintain protein structure. Metolazone, however, appears less effective based on these criteria.

These findings collectively demonstrate the universality and effectiveness of MTSSMol in accelerating the identification of potential drug candidates, solidifying its value as a practical tool for drug discovery. By integrating various experimental results, MTSSMol has demonstrated its exceptional ability to swiftly screen and identify candidate drugs, further proving its importance and reliability in pharmaceutical research. This discovery not only offers new avenues for drug development but also significantly enhances the efficiency of the drug discovery process.

### 3.4 Identifying HPK1 Inhibitors Using MTSSMol

To demonstrate the generalization of MTSSMol, we extend its application to the prediction of inhibitors targeting HPK1, a kinase that plays a critical role in immune regulation and is a promising target for drug discovery. We utilize a dataset of 4442 molecules with experimentally determined anti‐HPK1 activity, sourced from previous studies.^[^ ^47^ ^]^ To evaluate MTSSMol's performance comprehensively, we compare it with 11 baseline models based on self‐supervised learning, which include widely recognized frameworks for molecular property prediction.

As shown in Figure S11 (Supporting Information), MTSSMol consistently outperforms 11 baseline methods in terms of Spearman's r and Pearson's r, demonstrating its superior ability to capture the relationship between molecular features and inhibitory activity. These results highlight MTSSMol's robustness and reliability in predicting inhibitors with high precision. Moreover, this analysis confirms that MTSSMol's framework, trained on a diverse dataset of drug‐like molecules, is capable of generalizing to new and specific protein targets. By demonstrating strong performance across different predictions of target inhibitors, MTSSMol offers a versatile and powerful tool for accelerating the discovery of novel inhibitors in various therapeutic areas.

### 3.5 Ablation Analysis of MTSSMol

To validate the effectiveness of MTSSMol's design choices, we conduct a comprehensive ablation study by introducing several modified frameworks with specific limitations: MTSSMol‐NP (no pre‐training), MTSSMol‐MLCT (only multi‐label classification tasks), and MTSSMol‐MCL (only contrastive learning tasks). Table S10 (Supporting Information) presents the performance results on our benchmark dataset. These results show that MTSSMol achieves AUC scores that are 2.7% and 3.2% higher than those of MTSSMol‐MLCT and MTSSMol‐MCL, respectively. This indicates that combining two pre‐training strategies in MTSSMol significantly enhances information capture compared to using a single strategy. Additionally, MTSSMol demonstrates a 4.8% overall improvement compared to MTSSMol‐NP. In summary, by combining multi‐label classification and mask‐based contrastive learning, MTSSMol effectively captures multi‐level structural similarities through clustering and pseudo‐labeling while simultaneously enhancing robustness and structural awareness through masked graph contrastive learning. This dual approach allows the model to better utilize unlabeled molecular data and learn comprehensive molecular representations, leading to superior performance across diverse molecular property prediction tasks.

## 4 Discussion and Conclusion

In this study, we propose MTSSMol, a self‐supervised learning framework designed to provide a highly adaptive and robust molecular property prediction system through enhanced molecular representations and multi‐task pre‐training strategies. Our framework is rigorously validated across benchmark biomedical datasets encompassing a diverse range of drug discovery tasks, demonstrating superior performance. Notably, MTSSMol exhibits significant practical applications, particularly in identifying potential inhibitors of FGFR1, an important target in cancer therapy. The versatility and effectiveness of MTSSMol are further corroborated by molecular dynamics simulations, which provide an additional layer of validation. Our study highlights MTSSMol's potential to accelerate drug discovery processes, enhance predictive modeling, and offer insights into molecular interactions, thereby contributing to the development of novel therapeutics. Additionally, although its effectiveness in predicting FGFR1 inhibitors has been validated through molecular dynamics simulations, further experimental validation is needed to confirm its applicability in actual drug development.

Compared to SOTA methods, MTSSMol shows several improvements. First, MTSSMol excels in various drug discovery tasks such as assessing drug properties (e.g., blood‐brain barrier permeability, drug metabolism, and toxicity) and predicting molecular targets (e.g., FGFR1). Second, MTSSMol outperforms existing methods based on sequence, fingerprint, and graph‐based representations. Lastly, MTSSMol offers enhanced interpretability, enabling more intuitive recognition of biological‐relevant chemical structures or substructures involved in molecular property recognition and target binding.

Despite MTSSMol's advantages in effectively predicting molecular properties, there are still some limitations. First, the current pre‐training framework heavily relies on a large amount of unlabeled data, which may pose challenges in data acquisition and processing, especially for specific or rare compounds. Second, while MTSSMol excels in multiple drug discovery tasks, its performance may be influenced by dataset characteristics and model structure choices, necessitating further optimization and validation. Third, the current model's interpretability and transferability need improvement to ensure its universality and accuracy across different compounds and biological contexts. While the computational results underscore the potential utility of MTSSMol in early‐stage drug discovery, it is important to recognize that, although molecular docking and simulation methods are highly sophisticated, they cannot fully replace experimental validation, such as in vitro or in vivo assays, which remain essential to confirm the practical efficacy and biological relevance of the predicted compounds. Future research directions could explore integrating additional types of information and knowledge, such as richer molecular descriptors or 3‐D molecular conformational information, to enhance MTSSMol's representation learning capabilities and application scope. In conclusion, MTSSMol represents a promising self‐supervised learning framework but requires further development and optimization to achieve widespread application and long‐term sustainability in drug discovery.

## Conflict of Interest

The authors declare no conflict of interest.

## Author Contributions

X.Y. and Y.W. contributed equally to the paper as first authors. X.Y. performed data curation, investigation, methodology, and software, and wrote the original draft. Y.W. performed data curation, formal analysis, investigation, validation, and visualization, and wrote reviewed, and edited the original draft. Y.L. performed investigation and visualization. M.Z. performed investigation and visualization. O.L. performed investigation and visualization. J.S. performed conceptualization, funding acquisition, methodology, project administration, and supervision, and wrote reviewed, and edited the original draft. Q.Z. performed conceptualization, funding acquisition, methodology, project administration, and supervision, wrote reviewed, and edited the original draft.

## Supporting information



Supporting Information

## Data Availability

The source codes of MTSSMol are available online at https://github.com/zhaoqi106/MTSSMol. The datasets from the TDC benchmark platform are available at https://tdcommons.ai/. The FDA dataset is available at https://go.drugbank.com/releases/5‐1‐10/downloads/approvedstructure‐links. The reference protein‐ligand complex structures for FGFR1 used in this study are available in Protein Data Bank under accession codes 5A4C https://www.rcsb.org/structure/5A4C.
