# Supplementary material for: A Multi‐Task Self‐Supervised Strategy for Predicting Molecular Properties and FGFR1 Inhibitors
Source: Adv Sci (Weinh). 2025 Feb 8;12(13):2412987. doi: 10.1002/advs.202412987 (PMC11967764; doi:10.1002/advs.202412987)
Supplement: Supplementary file 1 — Supporting Information [file ADVS-12-2412987-s001.docx]

1. **Supplementary notes**
2. **Pre-training task details**

For the pre-training of MTSSMol, we utilize approximately 10 million unlabeled molecules from PubChem. Subsequently, we employ RDKit to construct molecular graphs and extract chemical features from SMILES strings. Each node and edge in the molecular graph represent atoms and bonds within a compound molecule. The pre-training dataset is randomly split into training and validation sets at a ratio of 95:5.

1. **Fine-tuning process**

After completing the pre-training of MTSSMol, we further enhance the performance of model through fine-tuning for downstream tasks. We add a randomly initialized MLP on top of the base GNNs feature extractor. CrossEntropyLoss is used for classification tasks, while L1Loss and MSELoss are used for regression tasks. For each task, we fine-tune the pre-trained model three times independently to obtain the average performance and standard deviation on the test set. The entire framework is implemented based on Pytorch Geometric.

1. **Downstream task details**

To comprehensively evaluate the performance of MTSSMol, we use random split to divide the datasets. Each dataset is split into training, validation, and test sets in 8:1:1 ratio. For the classification tasks, we use the area under the receiver operating characteristic AUC curve to evaluate classification tasks. In contrast, for regression tasks, we employ root mean square error (RMSE) or mean absolute error (MAE) as evaluation metrics. In FGFR1 inhibitors prediction task, the evaluation metric used are Spearman’s rank correlation coefficient (Spearman’s r) and Pearson correlation coefficient (Pearson’s r). The results are reported as the average and variance of three independent runs.

1. **Downstream molecular property datasets**

We employ three benchmarks in our tests to comprehensively evaluate the predictive performance of MTSSMol. MoleculeNet [1] is a popular benchmark for molecular property prediction. Here, we use 6 classification datasets (BBBP, Tox21, ClinTox, HIV, BACE, SIDER) and 5 regression datasets (FreeSolv, ESOL, Lipophilicity, QM7 and QM8) from MoleculeNet to evaluate MTSSMol. All details for datasets are provided in Table S3. The details of these datasets are described as follows.

- BACE is a collection of 1513 molecules, providing binary labels of molecules which measure whether these molecules can act as the inhibitors of human β-secretase 1 (BACE-1) or not [2].
- ClinTox is a collection of 1478 drugs approved through the US Food and Drug Administration approved (FDA) and eliminated due to the toxicity issues during clinical trials, providing two binary classification tasks [3].
- HIV dataset contains 41127 records of whether the compound inhibits HIV replication for binary classification between active and inactive.
- Tox21 is a public database containing twelve toxicity binary labels for 7831 molecules.
- BBBP is a binary classification dataset that contains 2039 molecules, recording whether these molecules can penetrate the blood-brain barrier or not [4].
- SIDER is a database of marketed drugs and adverse drug reactions (ADR). The version of the SIDER dataset in DeepChem classifies drug side effects into 27 system organ classes according to MedDRA's classification of 1427 approved drugs [5].
- FreeSolv dataset is a collection of experimental and calculated hydration free energies and their experimental values for small molecules in water [6].
- ESOL dataset is a regression dataset containing structures and water solubility data of compounds [7].
- Lipophilicity dataset collected from the ChEMBL database provides experimental results for 4200 compounds with respect to the octanol/water distribution coefficient (logD at pH 7.4), which is an important feature of drug molecules affecting membrane permeability and solubility [8].
- QM7 is a subset of GDB-13 (a database of nearly 1 billion stable and synthesizable organic molecules) that records the calculated atomization energies of stable and synthesizable organic molecules, such as HOMO/LUMO, atomization energies, etc. It contains various molecular structures (such as triple bonds, cycles, amides and epoxy resins) and up to 7 heavy atoms C, N, O, and S.
- QM8 dataset contains approximately 21786 data points, each representing a small organic molecule with quantum mechanical properties such as excitation and transition energies.

The second benchmark is Therapeutics Data Commons (TDC) benchmark [9]. The detailed information for these datasets from TDC benchmark is listed below:

- Absorption datasets
  - Pgp contains 1212 activity labels of P-glycoprotein (Pgp) inhibition, providing one binary classification task [10].
  - Bioav is a collection of 640 molecules with labels measuring the activity of bioavailability, providing one binary classification task [11].
  - AqSol contains 9982 labels measuring the solubility of molecules, providing one regression task [12].
- Distribution datasets
  - BBB is a dataset measuring the activity of the blood-brain barrier (BBB) of 1975 molecules, providing one binary classification dataset [4].
  - PPBR is a dataset measuring the human plasma protein binding rates (PPBRs) of 1614 molecules, providing one regression dataset [9].
- Toxicity datasets
  - LD50 is a collection of 7385 molecules with labels measuring their acute toxicity, providing one regression dataset [13].
  - hERG is a dataset containing 648 molecules measuring whether each molecule is a human ether a-go-go related gene (hERG) blocker or not, providing one classification dataset [14].
  - Ames is a dataset containing 7255 molecules measuring whether each molecule is mutagenic or not, providing one binary classification dataset [15].
  - DILI is a dataset containing 475 molecules measuring whether each molecule can cause liver injury or not, providing one binary classification dataset [16].
- Distribution datasets
  - CYP1A2 is a dataset measuring CYP1A2 inhibition of 12099 molecules, providing one binary classification task [17].
  - CYP2C9 is a dataset measuring CYP2C9 inhibition of 12130 molecules, providing one binary classification task [18].
  - CYP2D6 is a dataset measuring CYP2D6 inhibition of 11881 molecules, providing one binary classification task [18].
  - CYP2C19 is a dataset measuring CYP2C19 inhibition of 11885 molecules, providing one binary classification task [17].
  - CYP3A4 is a dataset measuring CYP3A4 inhibition of 11536 molecules, providing one binary classification task [18].

1. **Brief introduction of the baseline methods for molecular property prediction**

In the first benchmark test, we compare 12 newly proposed methods that have demonstrated strong competitiveness and superior performance in molecular property prediction tasks. The details of these methods are listed below.

- D-MPNN is a graph neural work that is specifically designed for molecular property prediction [19].
- N-Gram Graph builds the representation for the graph by assembling the vertex embedding in short walks, which needs no training [20].
- MGSSL is graph representation learning methods based on self-supervised learning [21].
- GraphLoG pre-trains a GIN through learning the hierarchical prototypes upon graph embeddings [22].
- 3D Infomax pre-trains a principle neighbor aggregation network (PNA) via maximizing the mutual information between the 3D graph representations and the corresponding 2D graph representations [23].
- MolCLR: Molecular Contrastive Learning of Representations via Graph Neural Networks (GNNs), a self-supervised learning framework that leverages large unlabeled data (∼10M unique molecules) [24].
- GROVER conducts pre-training of a message-passing transformer by incorporating both node-level and graph-level tasks [25].
- DVMP is a novel molecular pre-training method that utilizes a dual-view representation of molecules based on GNNs and transformers [26].
- KPGT integrates a graph transformer specifically designed for molecular graphs and a knowledge-guided pre-training strategy, to fully capture both structural and semantic knowledge of molecules [9].
- GraphMAE introduces a masked graph autoencoder that conducts pre-training with a feature reconstruction strategy [27].
- MoleBERT integrates a node-level pre-training strategy named masked atoms modeling and a triplet masked contrastive learning strategy for graph-level pre-training [28].
- MSSL2drug is a multi-task self-supervised model designed to predict interactions among drugs, proteins, and diseases. We adapt its input and output for molecular property prediction, providing a benchmark for evaluation.

1. **Comparison methods for drug metabolism prediction**

We compare the proposed MTSSMol with sequence-based methods (RNN_LR, TRFM_LR, RNN_MLP, TRFM_MLP,RNN_RF, TRFM_RF [29] and CHEM-BERT [30]) and graph-based methods (MolCLR_GIN_, MolCLR_GCN_ [24] and GROVER [25]) with more evaluation metrics (Accuracy, ROC-AUC, AUPR, F1, precision, recall, kappa) to verify the advantages of MTSSMol. In sequence-based methods, SMILES transformer uses RNN (Recurrent Neural Network) [31] and TRFM (TRansForMer) [32] to extract molecular representations and we use LR (Logistic Regression) [33], MLP (Multi-Layer Perception) [34] and RF (Random Forest) [35] as classifiers for downstream tasks. CHEM-BERT applies a pre-training task of BERT [36] on 9 million unlabeled molecules SMILES selected from ZINC [37] database. In graph-based methods, GROVER is a self-supervised message passing transformer, which is pre-trained on 10 million unlabelled molecules with node-level, edge-level and graph-level tasks. MolCLR developes GIN (graph isomorphism network) [38] or GCN (graph convolutional network) [39] encoders to learn differentiable representations on large unlabeled data (~10 million unique molecules) with three molecule graph augmentations (atom masking, bond deletion, and subgraph removal).

1. **Comparison methods o**n **FGFR1 dataset**

To ensure a comprehensive and rigorous evaluation of MTSSMol, we select eleven representative baseline methods from the field of molecular representation learning and drug discovery. These methods are chosen based on their state-of-the-art performance, widespread adoption, and conceptual relevance to our proposed framework. The specific reasons for selecting each baseline are as follows: KPGT [9], Contextpred [40], Infomax [41], JOAO [42], GraphMAE [27], Edgepred [43], Masking [40], GraphLoG [22], MolBERT [28], GraphMVP [44] and GraphCL [42]. These models collectively cover a diverse set of methodological paradigms, including contrastive learning, autoencoders, knowledge-based methods, and transformer-based architectures. By benchmarking MTSSMol against these baselines, we ensure a comprehensive performance evaluation that highlights the strengths and improvements brought by our proposed multi-task self-supervised framework in the context of molecular property prediction and drug discovery.

1. **Molecular dynamics methods**
2. **All-atom molecular dynamics simulation**

The predicted FGFR1 and ligand complex structure is solvated in a water box with 9 × 9 × 9 nm³ dimensions. CHARMM-GUI server generates the configuration, topology, and parameter files of all-atom molecular dynamics simulation systems using CHARMM36m force field [45-47]. The forcefield parameter of the ligand is generated by CgenFF [48]. Besides the predicted FGFR1 and ligand complex structure, the simulation system includes approximately 22600 water molecules, 69 sodium ions, and 64 chloride ions to mimic a 150 mM NaCl solution, resulting in a total of 70000 atoms. The all-atom molecular dynamics (MD) simulation is conducted using GROMACS 2023 at a temperature of 303 K with a time step of 2 fs [49]. Cubic periodic boundary conditions are applied, and van der Waals interactions are smoothly switched off between 1 and 1.2 nm. Long-range electrostatic interactions are calculated using particle mesh Ewald method. Energy minimization is performed using the steepest descent algorithm, followed by a 0.4 ns NVT (constant number of particles, volume, and temperature) equilibration and a 20 ns NPT (constant number of particles, pressure, and temperature) equilibration. During the equilibration, force restraints are gradually decreased from 1000 kJ mol⁻¹ nm⁻² to 400 kJ mol⁻¹ nm⁻² in NVT stage and from 400 kJ mol⁻¹ nm⁻² to 40 kJ mol⁻¹ nm⁻² in NPT stage. After equilibration, all force restraints are removed. The 100 ns unbiased MD simulation trajectories are carried out in NPT ensemble starting from the equilibrated predicted FGFR1 and ligand complex structure.

1. **Binding free energy calculation**

The binding free energy ∆G=RT lnk_d, where R is the gas constant, T is the absolute temperature, k_d is the dissociation constant. This formula can convert experimentally measured association or dissociation constants into binding free energy. Binding free energy predictions can also be obtained through MD simulations and free energy perturbation (FEP) calculations. FEP method is a widely used computational technique for predicting the free energy of interactions between molecules. By perturbing the state of system, it calculates the free energy differences between different states, thus predicting the binding free energy. These theoretically calculated binding free energy values can be compared with experimental measurements to validate the accuracy of computational models.

gmx_MMPBSA program is used to estimate the binding free energy between the ligand and receptor [50]. The binding energy is calculated by the following formula ∆G=∆H-T∆S, where ∆G is the binding energy between the ligand and receptor, ∆H is the enthalpy between the ligand and receptor, and T∆S represents interaction entropy (IE) between the ligand and receptor. The input file of gmx_MMPBSA contains all the parameters needed for MM/GBSA calculation of protein-protein interaction. In this case, all the frames are used when performing MM/GBSA calculation with the igb2 (GB-OBC1) model and a salt concentration of 150 mM. IE is calculated and the average of total number of frames is reported. Per-residue decamp with EEL and VDW potential terms is performed, and residues within 3Å in both receptor and ligand are printed in the output file.

1. **Supplementary Figure**

**Figure S1.** Confusion matrices for six molecular property prediction datasets based on random splitting. The results are obtained from the best model in three runs.


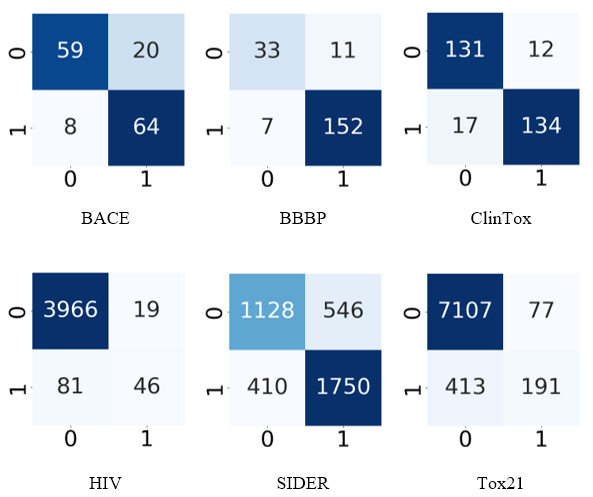


**Figure S2.** Confusion matrices for six TDC datasets based on random splitting. The results are obtained from the best model in three runs.


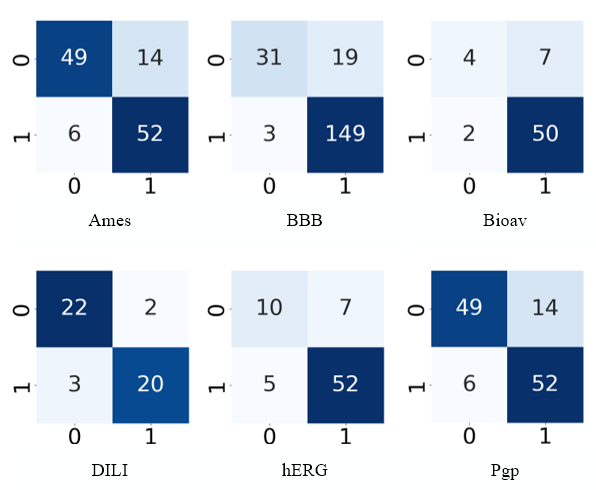


**Figure S3.** The confusion matrix on five CYP450 datasets with balanced scaffold split. The results are obtained from the best model in three runs.
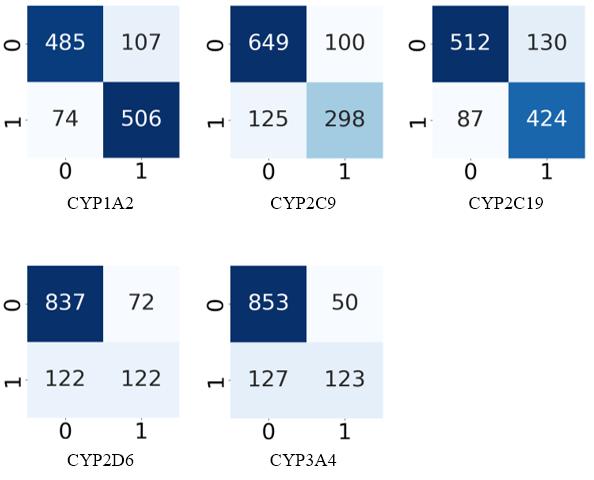


**Figure S4.** The AUROC curves on five CYP450 datasets with balanced scaffold split. 1st AUC, 2nd AUC and 3rd AUC represent the results of the first, second and third random runs, respectively. Avg AUC means macroaveraged AUC on three random runs.


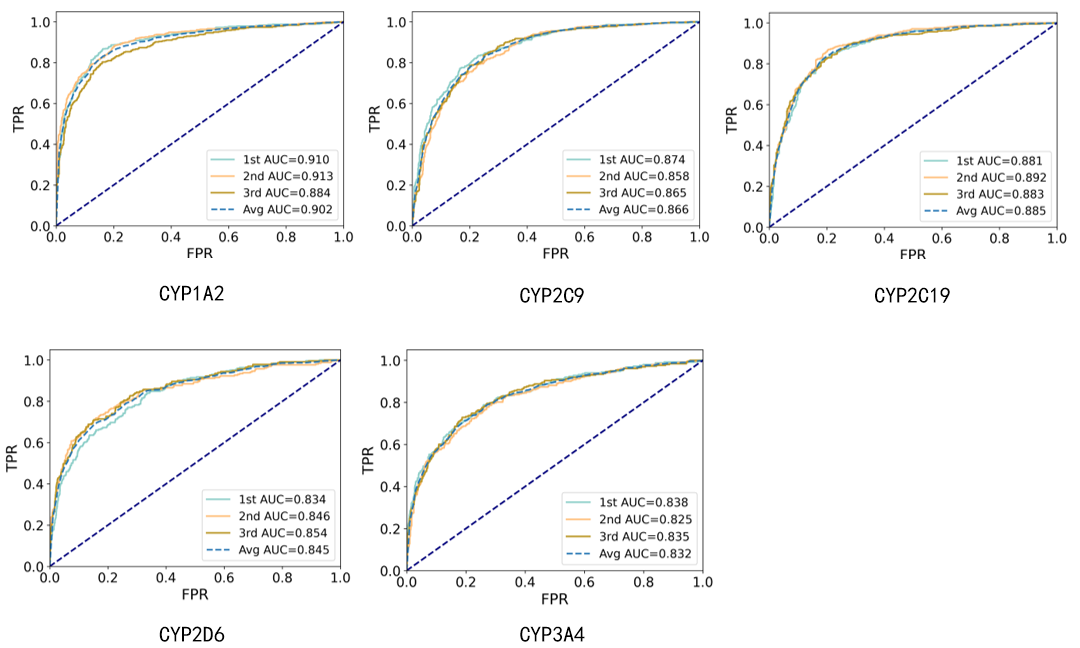


**Figure S5.** Pearman`s r between five descriptors (i.e., MolLogP, MolWt, TPSA, NumRotatableBonds, QED, and SA).


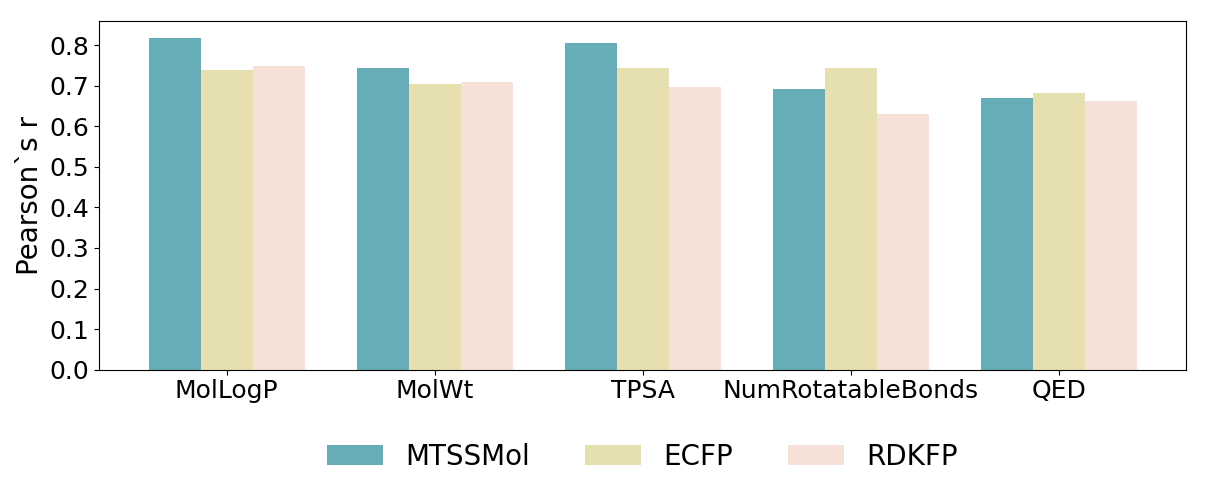


**Figure S6.** Activity cliffs identified by SubgraphX. Dashed circles highlight the distinguished substructures within the activity cliffs.

**

**

**Figure S7.** RMSD of the protein backbone over time for each inhibitor. This panel shows the stability of the protein-inhibitor complex during the molecular dynamic simulations.

**
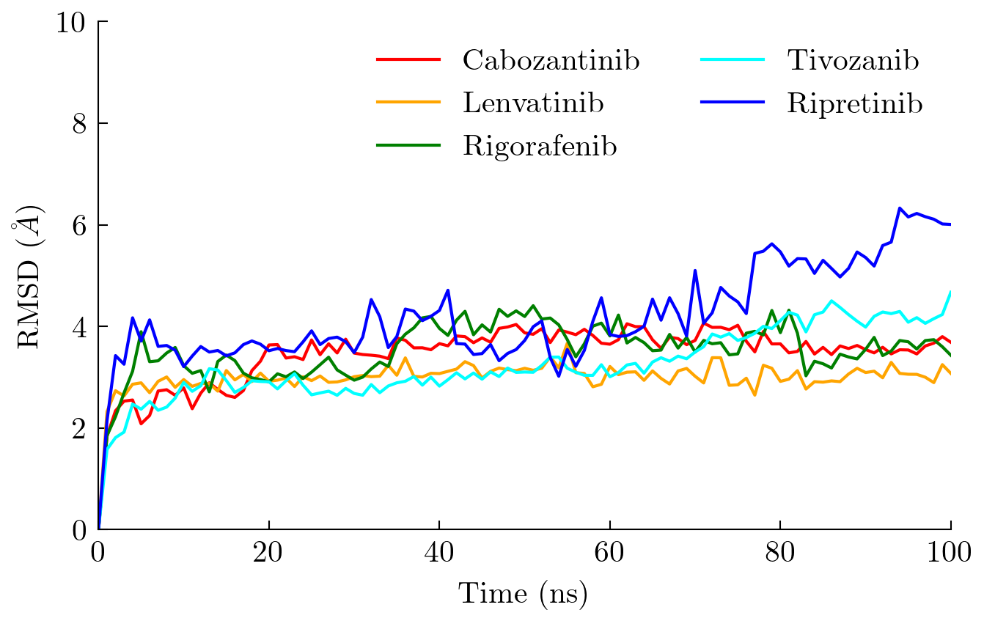
**

**Figure S8.** RMSD of the inhibitors themselves over time. This panel highlights the conformational changes of the inhibitors within the binding site throughout the simulations.


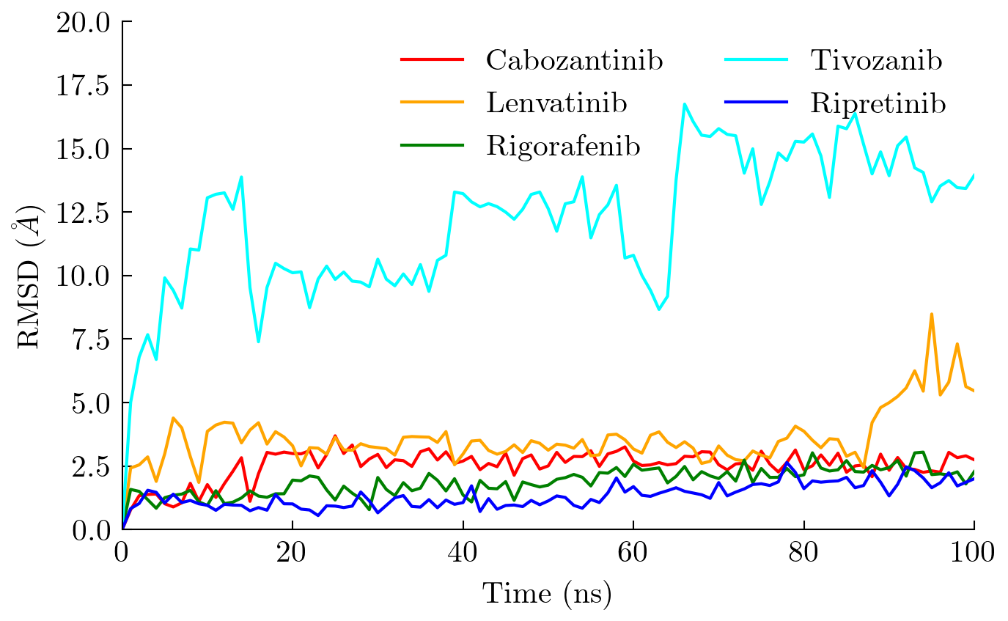


**Figure S9.** RMSD of the protein backbone atoms over the simulation time for each ligand. This plot shows the stability of the protein structure when bound to different ligands.

**
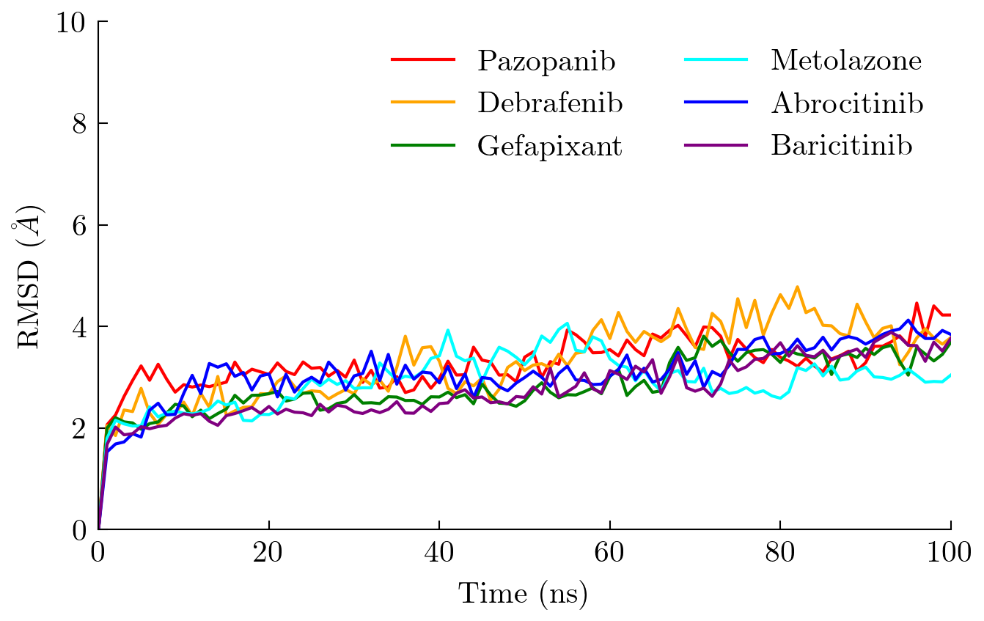
**

**Figure S10.** RMSD of the ligand heavy atoms over the simulation time. This indicates the stability of the ligand conformation within the binding pocket.

**
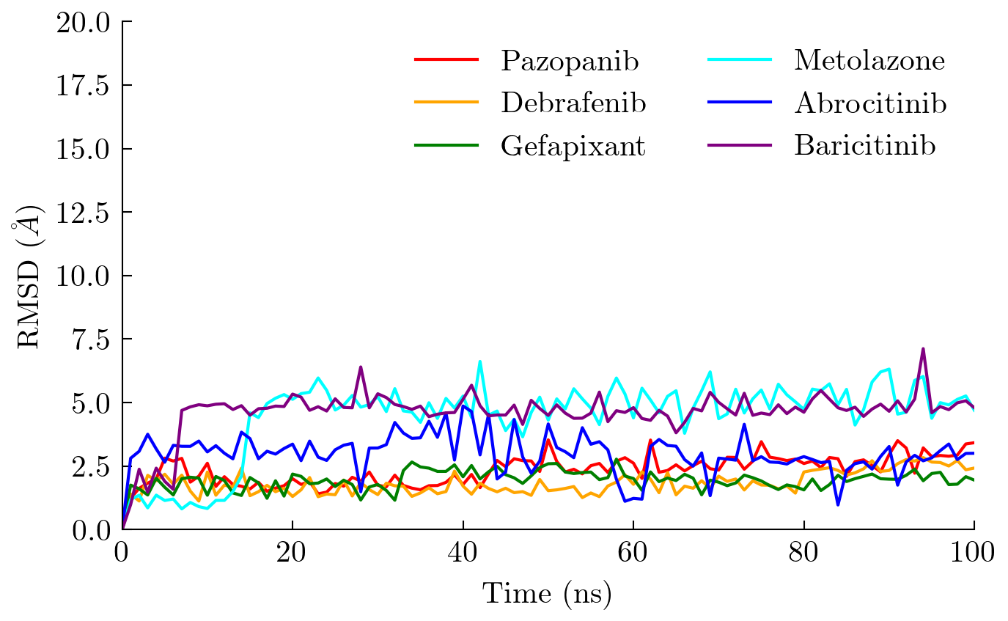
**

**Figure S11.** The performance of MTSSMol and baseline methods in predicting HPK1 inhibitors is measured by Pearson's r and Spearman's r, respectively.

**

**

1. **Supplementary Table**

**Table S1.** Node and edge features used in MTSSMol.

| Feature type | Feature name | Range |
| --- | --- | --- |
| Node feature | Atomic number  Chirality | [1, 119]  {unspecified, tetrahedral CW, tetrahedral CCW, other} |
| Edge feature | Bond type  Bond direction | {single, double, triple, aromatic}  {none, end-upright, end-downright} |

**Table S2.** The hyper-parameter settings of MTSSMol in the pre-training and finetuning process.

| Hyper-parameter | Pre-training | Finetuning |
| --- | --- | --- |
| Hidden size | 512 | 512 |
| Number of layers | 5 | 5 |
| Dropout rate | 0 | {0, 0.1, 0.2, 0.3} |
| Batch size | 512 | 32 |
| Learning rate | 0.00001 | {1e-4,5e-4, 1e-5, 5e-5} |
| Weight decay | le-5 | {0, le-5, le-6} |
| Predictor layer | NA | 3 |
| Masking rate | 0.25 | NA |
| Relnit top-n layer | NA | {1, 2, 3,4 } |
| FLAG step size | NA | {5e-3, 3e-3, 1e-2, 1e-3} |
| L2-SP weight | NA | {1e-1,1e-2,1e-3,1e-4} |
| valid_size | 0.05 | 0.1 |
| test_size | NA | 0.1 |
| splitting | NA | Random |

**Table S3.** The first benchmark contains datasets that have been widely used to evaluate the predictive performance of self-supervised learning methods on molecules, including six classification datasets and five regression datasets.

| Dataset | Molecules | Tasks | Task type | Metric |
| --- | --- | --- | --- | --- |
| BACE | 1513 | 1 | Classification | AUROC |
| BBBP | 2039 | 1 | Classification | AUROC |
| ClinTox | 1478 | 2 | Classification | AUROC |
| HIV | 41127 | 1 | Classification | AUROC |
| SIDER | 1427 | 27 | Classification | AUROC |
| Tox21 | 7831 | 12 | Classification | AUROC |
| FreSolv | 642 | 1 | Regression | RMSE |
| ESOL | 1128 | 1 | Regression | RMSE |
| Lipo | 4200 | 1 | Regression | RMSE |
| QM7 | 6830 | 1 | Regression | MAE |
| QM8 | 21786 | 12 | Regression | MAE |

**Table S4.** The AUROC performance of different methods on classification datasets in fine-tuning mode is

represented by the average ± standard deviation of three independent runs. "-" indicates that the

corresponding baseline algorithm is not evaluated on the dataset due to incompatibility with the dataset

type or task. The best result for each dataset is marked in bold.

| Method | BACE | BBBP | ClinTox | HIV | SIDER | Tox21 |
| --- | --- | --- | --- | --- | --- | --- |
| MTSSMol_GIN_ | **0.915±0.008** | **0.930±0.022** | 0.842±0.028 | **0.834±0.005** | 0.643±0.009 | **0.857±0.005** |
| MTSSMol_GAT_ | 0.891±0.024 | 0.885±0.027 | 0.816±0.015 | 0.771±0.015 | 0.642±0.005 | 0.801±0.015 |
| MTSSMol_GCN_ | 0.885±0.011 | 0.915±0.029 | 0.839±0.041 | 0.787±0.013 | 0.622±0.021 | 0.828±0.009 |
| D-MPNN | 0.853±0.053 | 0.712±0.038 | 0.905±0.053 | 0.750±0.021 | 0.632±0.023 | 0.689±0.013 |
| N-GramRF | 0.779±0.015 | 0.697±0.006 | 0.775±0.04 | 0.772±0.001 | 0.668±0.007 | 0.743±0.004 |
| N-GramXGB | 0.791±0.013 | 0.691±0.008 | 0.875±0.027 | 0.787±0.004 | 0.655±0.007 | 0.758±0.009 |
| MGSSL | 0.791±0.009 | 0.705±0.011 | 0.807±0.021 | 0.795±0.011 | 0.618±0.008 | 0.765±0.003 |
| GraphLoG | 0.835±0.012 | 0.725±0.008 | 0.767±0.033 | 0.778±0.008 | 0.612±0.011 | 0.765±0.003 |
| 3D Infomax | 0.794±0.019 | 0.691±0.011 | 0.594±0.032 | 0.761±0.013 | 0.534±0.033 | 0.745±0.007 |
| MolCLR | 0.89±0.003 | 0.738±0.002 | 0.932±0.017 | 0.806±0.011 | 0.68±0.011 | 0.798±0.007 |
| GROVER | 0.894±0.028 | 0.885±0.009 | 0.944±0.021 | - | 0.658±0.023 | 0.831±0.025 |
| DVMP | 0.894±0.008 | 0.778±0.003 | **0.956±0.007** | 0.814±0.004 | **0.698±0.006** | 0.791±0.004 |
| KPGT | 0.855±0.011 | 0.908±0.01 | 0.946±0.022 | - | 0.649±0.009 | 0.838±0.013 |
| GraphMAE | 0.831±0.009 | 0.72±0.006 | 0.823±0.012 | 0.782±0.008 | 0.603±0.011 | 0.755±0.006 |
| Mole-BERT | 0.808±0.014 | 0.719±0.016 | 0.789±0.03 | - | 0.628±0.011 | 0.768±0.005 |
| MSSL2drug | 0.781±0.12 | 0.716±0.011 | 0.796±0.016 | 0.741±0.005 | 0.613±0.006 | 0.751±0.008 |

**Table S5.** The average RMSE (FreeSolv, ESOL, Lipo) or MAE (QM7, QM8) performance of different methods on regression datasets in fine-tuning mode is represented by the average ± standard deviation of three independent runs. "-" indicates that the corresponding baseline algorithm is not evaluated on the dataset due to incompatibility with the dataset type or task. The best result for each dataset is marked in bold.

| Method | FreSolv | ESOL | Lipo | QM7 | QM8 |
| --- | --- | --- | --- | --- | --- |
| MTSSMol_GIN_ | **1.209±0.052** | **0.798±0.046** | 0.65±0.019 | **67.4±1.5** | **0.0125±0.0003** |
| MTSSMol_GAT_ | 1.924±0.024 | 1.016±0.058 | 0.746±0.075 | 79.4±4.2 | 0.0128±0.0012 |
| MTSSMol_GCN_ | 1.961±0.184 | 0.906±091 | 0.743±9.008 | 77.31±4.5 | 0.0152±0.0004 |
| D-MPNN | 2.082±0.082 | 1.050±0.008 | 0.683±0.016 | 103.5±8.6 | 0.0190±0.0001 |
| N-GramRF | 2.688±0.085 | 1.074±0.107 | 0.812±0.028 | 92.8±4.0 | 0.0236±0.0006 |
| N-GramXGB | 5.061±0.744 | 1.083±0.082 | 2.072±0.030 | 81.9±1.9 | 0.0215±0.0005 |
| 3D Infomax | 2.337±0.227 | 0.894±0.028 | 0.695±0.012 | - | - |
| MolCLR | 2.200±0.200 | 1.110±0.010 | 0.650±0.080 | 87.2±2.0 | 0.0174±0.0013 |
| GROVER | 1.544±0.397 | 0.831±0.120 | **0.560±0.035** | 72.6±3.8 | 0.0125±0.002 |
| DVMP | 1.952±0.061 | 0.817±0.024 | 0.653±0.002 | 74.4±1.2 | 0.0171±0.0004 |
| KPGT | 2.314±0.841 | 0.848±0.0103 | 0.656±0.023 | - | - |
| GraphMAE | 3.023±0.779 | 1.378±0.028 | 0.746±0.014 | - | - |
| Mole-BERT | 2.801±0.062 | 1.015±0.030 | 0.676±0.017 | - | - |
| MSSL2drug | 2.93±0.12 | 1.32±0.02 | 0.85±0.07 | 110.2±6.4 | 0.0223±0.0021 |

**Table S6.** MTSSMol's predictive performance on TDC benchmark dataset, presented as the average ± standard deviation of three independent runs, along with the best baseline methods provided by TDC benchmark leaderboard. The best result for each dataset is marked in bold.

| Dataset description | | | | Current best baseline | | | MTSSMol |
| --- | --- | --- | --- | --- | --- | --- | --- |
| Group | Dataset | Task | Metric | Method | Type | Score | Score |
| Absorption | Pgp | Classification | AUROC | KPGT | DL | 0.938±0.004 | **0.941±0.017** |
|  | Bioav | Classification | AUROC | KPGT | DL | **0.750±0.022** | 0.731±0.08 |
|  | AqSol | Regression | MAE | KPGT | DL | 0.714±0.011 | **0.698±0.003** |
| Distribution | BBB | Classification | AUROC | LRE | DL | 0.908±0.005 | **0.933±0.002** |
|  | PPBR | Regression | MAE | KPGT | DL | 7.684±0.250 | **7.21±0.406** |
| Toxicity | LD50 | Regression | MAE | KPGT | DL | 0.545±0.010 | **0.431±0.02** |
|  | hERG | Classification | AUROC | SimGCN | DL | 0.874±0.014 | **0.880±0.03** |
|  | Ames | Classification | AUROC | ZairaChem | ML | 0.871±0.002 | **0.873±0.004** |
|  | DILI | Classification | AUROC | KPGT | DL | 0.929±0.013 | **0.933±0.006** |

**Table S7.** The performance of different methods on CYP450 datasets, presented as the average ± standard deviation of three independent runs. The best result for each dataset is marked in bold.

|  | | | | | | | |
| --- | --- | --- | --- | --- | --- | --- | --- |
| CYP1A2 | | | | | | | |
| Methods | Accuracy | AUC | AUPRC | F1 | Precision | Recall | Kappa |
| **MTSSMol** | **0.832±0.010** | **0.902±0.012** | **0.907±0.011** | **0.829±0.014** | 0.827±0.003 | 0.831±0.032 | **0.664±0.021** |
| MolCLR_GIN_ | 0.831±0.005 | 0.900±0.003 | 0.893±0.007 | 0.829±0.004 | 0.818±0.008 | **0.841±0.006** | 0.661±0.009 |
| MolCLR_GCN_ | 0.814±0.007 | 0.885±0.001 | 0.871±0.002 | 0.803±0.009 | 0.825±0.004 | 0.782±0.018 | 0.626±0.014 |
| RNN_LR | 0.722±0.000 | 0.790±0.000 | 0.759±0.000 | 0.707±0.000 | 0.724±0.000 | 0.691±0.000 | 0.443±0.000 |
| TRFM_LR | 0.791±0.000 | 0.868±0.000 | 0.869±0.000 | 0.778±0.000 | 0.804±0.000 | 0.754±0.000 | 0.581±0.000 |
| RNN_MLP | 0.681±0.023 | 0.771±0.007 | 0.739±0.007 | 0.631±0.061 | 0.715±0.017 | 0.576±0.115 | 0.357±0.050 |
| TRFM_MLP | 0.774±0.004 | 0.850±0.002 | 0.857±0.003 | 0.754±0.011 | 0.803±0.022 | 0.712±0.037 | 0.546±0.008 |
| RNN_RF | 0.780±0.003 | 0.855±0.002 | 0.856±0.002 | 0.764±0.004 | 0.797±0.003 | 0.733±0.004 | 0.558±0.006 |
| TRFM_RF | 0.790±0.004 | 0.871±0.000 | 0.871±0.000 | 0.778±0.004 | 0.799±0.005 | 0.758±0.004 | 0.578±0.007 |
| CHEM-BERT | 0.794±0.006 | 0.874±0.004 | 0.871±0.007 | 0.772±0.008 | **0.834±0.021** | 0.720±0.022 | 0.586±0.013 |
| GROVER | 0.809±0.001 | 0.884±0.000 | 0.882±0.000 | 0.798±0.002 | 0.823±0.006 | 0.774±0.007 | 0.618±0.002 |
|  |  |  |  |  |  |  |  |
| CYP2C9 | | | | | | | |
| Methods | Accuracy | AUC | AUPRC | F1 | Precision | Recall | Kappa |
| **MTSSMol** | **0.790±0.013** | **0.866±0.007** | **0.775±0.017** | 0.686±0.030 | **0.762±0.020** | 0.627±0.060 | **0.531±0.034** |
| MolCLR_GIN_ | 0.779±0.007 | 0.838±0.008 | 0.724±0.016 | **0.706±0.012** | 0.694±0.016 | **0.719±0.031** | 0.529±0.016 |
| MolCLR_GCN_ | 0.757±0.003 | 0.818±0.002 | 0.714±0.002 | 0.651±0.016 | 0.690±0.012 | 0.617±0.036 | 0.465±0.013 |
| RNN_LR | 0.698±0.000 | 0.743±0.000 | 0.615±0.000 | 0.522±0.000 | 0.627±0.000 | 0.447±0.000 | 0.310±0.000 |
| TRFM_LR | 0.720±0.000 | 0.780±0.000 | 0.682±0.000 | 0.603±0.000 | 0.630±0.000 | 0.579±0.000 | 0.387±0.000 |
| RNN_MLP | 0.653±0.012 | 0.729±0.007 | 0.578±0.009 | 0.401±0.157 | 0.587±0.058 | 0.393±0.285 | 0.199±0.078 |
| TRFM_MLP | 0.727±0.008 | 0.776±0.006 | 0.668±0.004 | 0.625±0.012 | 0.634±0.014 | 0.616±0.019 | 0.411±0.017 |
| RNN_RF | 0.719±0.001 | 0.763±0.000 | 0.623±0.002 | 0.603±0.003 | 0.628±0.001 | 0.580±0.005 | 0.386±0.003 |
| TRFM_RF | 0.735±0.001 | 0.798±0.001 | 0.691±0.002 | 0.614±0.001 | 0.664±0.003 | 0.571±0.001 | 0.414±0.002 |
| CHEM-BERT | 0.752±0.006 | 0.822±0.003 | 0.711±0.002 | 0.649±0.019 | 0.681±0.033 | 0.627±0.065 | 0.458±0.009 |
| GROVER | 0.745±0.005 | 0.814±0.002 | 0.725±0.003 | 0.595±0.024 | 0.719±0.013 | 0.509±0.039 | 0.417±0.019 |
|  |  |  |  |  |  |  |  |
| CPY2C19 | | | | | | | |
| Methods | Accuracy | AUC | AUPRC | F1 | Precision | Recall | Kappa |
| **MTSSMol** | **0.814±0.006** | **0.885±0.005** | **0.856±0.011** | **0.799±0.004** | **0.778±0.009** | 0.822±0.009 | **0.627±0.011** |
| MolCLR_GIN_ | 0.789±0.010 | 0.865±0.000 | 0.833±0.002 | 0.787±0.006 | 0.741±0.019 | **0.840±0.012** | 0.579±0.020 |
| MolCLR_GCN_ | 0.792±0.001 | 0.862±0.001 | 0.837±0.002 | 0.785±0.002 | 0.755±0.009 | 0.819±0.015 | 0.584±0.002 |
| RNN_LR | 0.692±0.000 | 0.751±0.000 | 0.692±0.000 | 0.662±0.000 | 0.677±0.000 | 0.648±0.000 | 0.380±0.000 |
| TRFM_LR | 0.729±0.000 | 0.816±0.000 | 0.785±0.000 | 0.708±0.000 | 0.709±0.000 | 0.708±0.000 | 0.455±0.000 |
| RNN_MLP | 0.667±0.014 | 0.739±0.006 | 0.684±0.002 | 0.624±0.058 | 0.660±0.039 | 0.613±0.124 | 0.327±0.033 |
| TRFM_MLP | 0.725±0.010 | 0.789±0.005 | 0.755±0.004 | 0.696±0.016 | 0.716±0.007 | 0.678±0.026 | 0.445±0.022 |
| RNN_RF | 0.715±0.003 | 0.790±0.001 | 0.734±0.001 | 0.696±0.002 | 0.691±0.004 | 0.701±0.002 | 0.428±0.005 |
| TRFM_RF | 0.747±0.003 | 0.824±0.001 | 0.792±0.001 | 0.728±0.003 | 0.728±0.005 | 0.727±0.006 | 0.491±0.006 |
| CHEM-BERT | 0.780±0.006 | 0.855±0.001 | 0.821±0.007 | 0.771±0.011 | 0.747±0.012 | 0.798±0.031 | 0.560±0.014 |
| GROVER | 0.759±0.001 | 0.838±0.000 | 0.798±0.001 | 0.737±0.001 | 0.749±0.002 | 0.725±0.004 | 0.514±0.002 |
| CYP2D6 | | | | | | | |
| Methods | Accuracy | AUC | AUPRC | F1 | Precision | Recall | Kappa |
| **MTSSMol** | **0.847±0.11** | **0.845±0.008** | **0.663±0.032** | **0.591±0.029** | 0.686±0.041 | 0.520±0.024 | **0.500±0.034** |
| MolCLR_GIN_ | 0.812±0.004 | 0.823±0.001 | 0.621±0.002 | 0.583±0.003 | 0.576±0.012 | **0.591±0.016** | 0.452±0.003 |
| MolCLR_GCN_ | 0.816±0.003 | 0.819±0.001 | 0.623±0.004 | 0.505±0.015 | 0.680±0.023 | 0.403±0.024 | 0.408±0.014 |
| RNN_LR | 0.787±0.000 | 0.670±0.000 | 0.387±0.000 | 0.206±0.000 | 0.582±0.000 | 0.125±0.000 | 0.139±0.000 |
| TRFM_LR | 0.798±0.000 | 0.754±0.000 | 0.504±0.000 | 0.392±0.000 | 0.586±0.000 | 0.294±0.000 | 0.286±0.000 |
| RNN_MLP | 0.780±0.004 | 0.670±0.007 | 0.369±0.016 | 0.204±0.014 | 0.515±0.046 | 0.128±0.013 | 0.127±0.006 |
| TRFM_MLP | 0.782±0.007 | 0.721±0.003 | 0.465±0.010 | 0.448±0.002 | 0.511±0.021 | 0.400±0.012 | 0.316±0.008 |
| RNN_RF | 0.803±0.002 | 0.727±0.001 | 0.478±0.002 | 0.398±0.002 | 0.611±0.010 | 0.295±0.002 | 0.297±0.003 |
| TRFM_RF | 0.806±0.002 | 0.769±0.001 | 0.531±0.002 | 0.315±0.011 | **0.719±0.014** | 0.201±0.008 | 0.241±0.011 |
| CHEM-BERT | 0.813±0.011 | 0.780±0.014 | 0.550±0.032 | 0.483±0.012 | 0.632±0.064 | 0.395±0.030 | 0.376±0.016 |
| GROVER | 0.807±0.003 | 0.789±0.003 | 0.534±0.012 | 0.331±0.031 | 0.706±0.006 | 0.217±0.027 | 0.254±0.027 |
| CYP3A4 | | | | | | | |
| Methods | Accuracy | AUC | AUPRC | F1 | Precision | Recall | Kappa |
| **MTSSMol** | **0.840±0.005** | 0.833±0.006 | 0.655±0.024 | 0.564±0.016 | 0.706±0.007 | 0.470±0.023 | 0.471±0.018 |
| MolCLR_GIN_ | 0.833±0.003 | **0.898±0.001** | **0.821±0.006** | **0.746±0.010** | **0.749±0.015** | **0.745±0.033** | **0.622±0.009** |
| MolCLR_GCN_ | 0.810±0.004 | 0.874±0.001 | 0.770±0.003 | 0.686±0.011 | 0.755±0.001 | 0.629±0.018 | 0.551±0.012 |
| RNN_LR | 0.775±0.000 | 0.826±0.000 | 0.674±0.000 | 0.635±0.000 | 0.685±0.000 | 0.591±0.000 | 0.474±0.000 |
| TRFM_LR | 0.806±0.000 | 0.867±0.000 | 0.782±0.000 | 0.707±0.000 | 0.704±0.000 | 0.711±0.000 | 0.562±0.000 |
| RNN_MLP | 0.757±0.003 | 0.812±0.006 | 0.655±0.007 | 0.610±0.035 | 0.652±0.028 | 0.582±0.090 | 0.436±0.026 |
| TRFM_MLP | 0.782±0.005 | 0.844±0.004 | 0.713±0.015 | 0.667±0.004 | 0.673±0.014 | 0.662±0.010 | 0.506±0.009 |
| RNN_RF | 0.779±0.004 | 0.837±0.000 | 0.713±0.001 | 0.654±0.007 | 0.675±0.004 | 0.635±0.010 | 0.492±0.010 |
| TRFM_RF | 0.810±0.004 | 0.862±0.001 | 0.773±0.004 | 0.697±0.007 | 0.732±0.007 | 0.666±0.007 | 0.559±0.010 |
| CHEM-BERT | 0.827±0.004 | 0.892±0.004 | 0.808±0.009 | 0.745±0.014 | 0.729±0.025 | 0.766±0.053 | 0.615±0.013 |
| GROVER | 0.826±0.003 | 0.891±0.001 | 0.817±0.002 | 0.720±0.004 | 0.768±0.013 | 0.678±0.013 | 0.594±0.005 |

**Table S8.** Top 25 predicted by MTSSMol to be potential inhibitors against FGFR1 with known evidence found from previous studies in the literature.

| DrugBank ID | Name | Prediction (pIC50) | Evidence |
| --- | --- | --- | --- |
| DB12147 | Erdafitinib | 9.154024 | Erdafitinib inhibited FGFR1 in TR-FRET assay with IC50 value of 1.2 nmol/L [51]. |
| DB15149 | Futibatinib | 8.302047 | Futibatinib selectively inhibited FGFR1 with IC(50) values of 1.4 to 3.7 nmol/L [52] |
| DB06589 | Pazopanib | 7.7273054 | Pazopanib inhibited FGFR1 with IC50 value of 140 nM [53]. |
| DB11817 | Baricitinib | 7.694054 | Not found. |
| DB08881 | Vemurafenib | 7.6849837 | Vemurafenib inhibited FGFR1 in kinase inhibition assay with inhibition rate of 24% [54]. |
| DB08875 | Cabozantinib | 7.6238995 | Cabozantinib inhibited FGFR1 with IC50 value of 11.3 nM [55]. |
| DB08901 | Ponatinib | 7.564068 | Ponatinib inhibited FGFR1 in ELISA assay with IC50 value of 0.7 nmol/L [56]. |
| DB11886 | Infigratinib | 7.553592 | Infigratinib binds to FGFR1 with IC50 value of 1.1 nM [57]. |
| DB13874 | Enasidenib | 7.286674 | Not found. |
| DB09079 | Nintedanib | 7.229935 | Ninetedanib inhibited FGFR1 in ELISA assay with  value of 0.06 uM [58]. |
| DB15685 | Selpercatinib | 7.18903 | Selpercatinib exhibits an IC₅₀ of approximately 50–70 nM against FGFR1. [59] |
| DB11986 | Entrectinib | 7.0313168 | Entretinib inhibited FGFR1 with IC50 value of 1 uM [60]. |
| DB15102 | Pemigatinib | 7.004992 | Pemigatinib inhibited FGFR1 in FRET assay with  IC50 value of 0.4 nM [61]. |
| DB11718 | Encorafenib | 6.8102202 | Not found. |
| DB09078 | Lenvatinib | 6.755518 | Lenvatinib inhibited FGFR1 in tyrosine kinase assay with IC50 value of 46 nM [62]. |
| DB11979 | Elagolix | 6.654944 | Not found. |
| DB11800 | Tivozanib | 6.589039 | Not found. |
| DB15822 | Pralsetinib | 6.4782815 | Not found. |
| DB14840 | Ripretinib | 6.4777813 | Not found |
| DB06626 | Axitinib | 6.3274264 | Axitinib inhibited FGFR1 with Ki value of 0.2 nM [63]. |
| DB12010 | Fostamatinib | 6.2870717 | Fostamatinib can act as inhibitor of FGFR1 [64] |
| DB00398 | Sorafenib | 6.198518 | Not found. |
| DB08877 | Ruxolitinib | 6.1751986 | Not found. |
| DB08896 | Regorafenib | 6.156093 | Not found. |
| DB08865 | Crizotinib | 6.101434 | Crizotinib inhibited FGFR1 in ELISA with IC50 value  of 1000 nM [65]. |

**Table S9.** Results predicted by MTSSMol for each family of previously experimentally proven or unproven potential inhibitors, as well as MW and LogP values.

| Family | Drugbank_ID | | name | Prediction | MW | LogP |
| --- | --- | --- | --- | --- | --- | --- |
| 1 | DB08875 | Cabozantinib | | 7.6238995 | 501.51 | 5.54 |
|  | DB09078 | Lenvatinib | | 6.755518 | 426.86 | 4.07 |
|  | DB08896 | Regorafenib | | 6.156093 | 482.82 | 5.69 |
|  | DB11800 | Tivozanib | | 6.589039 | 454.87 | 5.64 |
|  | DB14840 | Ripretinib | | 6.4777813 | 510.37 | 5.67 |
| 2 | DB06589 | Pazopanib | | 7.7273054 | 418.46 | 3.14 |
|  | DB08912 | Debrafenib | | 5.7305365 | 519.57 | 5.36 |
|  | DB15097 | Gefapixant | | 6.3128476 | 353.4 | 1.21 |
|  | DB00524 | Metolazone | | 4.6145883 | 365.84 | 2.71 |
|  | DB11817 | Baricitinib | | 7.694054 | 437.53 | 1.1 |
|  | DB14973 | Abrocitinib | | 5.241482 | 323.42 | 1.25 |

**Table S10.** Comparison analysis of MTSSMol and its three variants on MoleculeNet dataset. The best result for each dataset is marked in bold.

| Method | Classification dataset  **AVG** (AUROC) | Regression dataset  **AVG** (RMSE) |
| --- | --- | --- |
| MTSSMol | **0.836** | **0.885** |
| MTSSMol-MLCT | 0.814 | 1.178 |
| MTSSMol-MCL | 0.81 | 1.025 |
| MTSSMol-NP | 0.798 | 1.397 |

1. **Supplementary References**

[1]Wu Z, Ramsundar B, Feinberg EN, Gomes J, Geniesse C, Pappu AS, Leswing K, Pande V. MoleculeNet: a benchmark for molecular machine learning. Chemical science, 2018, 9(2):513-530.

[2]Subramanian G, Ramsundar B, Pande V, Denny RA. Computational Modeling of β-Secretase 1 (BACE-1) Inhibitors Using Ligand Based Approaches. Journal of chemical information and modeling, 2016, 56(10):1936-1949.

[3]Gayvert KM, Madhukar NS, Elemento O. A Data-Driven Approach to Predicting Successes and Failures of Clinical Trials. Cell chemical biology, 2016, 23(10):1294-1301.

[4]Martins IF, Teixeira AL, Pinheiro L, Falcao AO. A Bayesian approach to in silico blood-brain barrier penetration modeling. Journal of chemical information and modeling, 2012, 52(6):1686-1697.

[5]Kuhn M, Letunic I, Jensen LJ, Bork P. The SIDER database of drugs and side effects. Nucleic acids research, 2016, 44(D1):D1075-1079.

[6]Mobley DL, Guthrie JP. FreeSolv: a database of experimental and calculated hydration free energies, with input files. Journal of computer-aided molecular design, 2014, 28(7):711-720.

[7]Delaney JS. ESOL: estimating aqueous solubility directly from molecular structure. Journal of chemical information and computer sciences, 2004, 44(3):1000-1005.

[8]Gaulton A, Bellis LJ, Bento AP, Chambers J, Davies M, Hersey A, Light Y, McGlinchey S, Michalovich D, Al-Lazikani B *et al*. ChEMBL: a large-scale bioactivity database for drug discovery. Nucleic acids research, 2012, 40(Database issue):D1100-1107.

[9]Li H, Zhang R, Min Y, Ma D, Zhao D, Zeng J. A knowledge-guided pre-training framework for improving molecular representation learning. Nature Communication, 2023, 14(1):7568.

[10]Broccatelli F, Carosati E, Neri A, Frosini M, Goracci L, Oprea TI, Cruciani G. A novel approach for predicting P-glycoprotein (ABCB1) inhibition using molecular interaction fields. Journal of medicinal chemistry, 2011, 54(6):1740-1751.

[11]Ma CY, Yang SY, Zhang H, Xiang ML, Huang Q, Wei YQ. Prediction models of human plasma protein binding rate and oral bioavailability derived by using GA-CG-SVM method. Journal of pharmaceutical and biomedical analysis, 2008, 47(4-5):677-682.

[12]Sorkun MC, Khetan A, Er S. AqSolDB, a curated reference set of aqueous solubility and 2D descriptors for a diverse set of compounds. Scientific data, 2019, 6(1):143.

[13]Zhu H, Martin TM, Ye L, Sedykh A, Young DM, Tropsha A. Quantitative structure-activity relationship modeling of rat acute toxicity by oral exposure. Chemical research in toxicology, 2009, 22(12):1913-1921.

[14]Wang S, Sun H, Liu H, Li D, Li Y, Hou T. ADMET Evaluation in Drug Discovery. 16. Predicting hERG Blockers by Combining Multiple Pharmacophores and Machine Learning Approaches. Molecular pharmaceutics, 2016, 13(8):2855-2866.

[15]Xu C, Cheng F, Chen L, Du Z, Li W, Liu G, Lee PW, Tang Y. In silico prediction of chemical Ames mutagenicity. Journal of chemical information and modeling, 2012, 52(11):2840-2847.

[16]Xu Y, Dai Z, Chen F, Gao S, Pei J, Lai L. Deep Learning for Drug-Induced Liver Injury. Journal of chemical information and modeling, 2015, 55(10):2085-2093.

[17]Zeng X, Xiang H, Yu L, Wang J, Li K, Nussinov R, Cheng F. Accurate prediction of molecular properties and drug targets using a self-supervised image representation learning framework. Nature Machine Intelligence, 2022, 4(11):1004-1016.

[18]Veith H, Southall N, Huang R, James T, Fayne D, Artemenko N, Shen M, Inglese J, Austin CP, Lloyd DG *et al*. Comprehensive characterization of cytochrome P450 isozyme selectivity across chemical libraries. Nature biotechnology, 2009, 27(11):1050-1055.

[19]Yang K, Swanson K, Jin W, Coley C, Eiden P, Gao H, Guzman-Perez A, Hopper T, Kelley B, Mathea M *et al*. Analyzing Learned Molecular Representations for Property Prediction. Journal of chemical information and modeling, 2019, 59(8):3370-3388.

[20]Liu S, Demirel MF, Liang Y. N-gram graph: Simple unsupervised representation for graphs, with applications to molecules. Advances in neural information processing systems, 2019, 32.

[21]Zhang Z, Liu Q, Wang H, Lu C, Lee C-K. Motif-based graph self-supervised learning for molecular property prediction. Advances in Neural Information Processing Systems, 2021, 34:15870-15882.

[22]Xu M, Wang H, Ni B, Guo H, Tang J. Self-supervised graph-level representation learning with local and global structure. International Conference on Machine Learning, 2021, PMLR:11548-11558.

[23]Stärk H, Beaini D, Corso G, Tossou P, Dallago C, Günnemann S, Liò P. 3d infomax improves gnns for molecular property prediction. International Conference on Machine Learning, 2022, PMLR:20479-20502.

[24]Wang Y, Wang J, Cao Z, Barati Farimani A. Molecular contrastive learning of representations via graph neural networks. Nature Machine Intelligence, 2022, 4(3):279-287.

[25]Rong Y, Bian Y, Xu T, Xie W, Wei Y, Huang W, Huang J. Self-supervised graph transformer on large-scale molecular data. Advances in neural information processing systems, 2020, 33:12559-12571.

[26]Zhu J, Xia Y, Wu L, Xie S, Zhou W, Qin T, Li H, Liu T-Y. Dual-view Molecular Pre-training. Proceedings of the 29th ACM SIGKDD Conference on Knowledge Discovery and Data Mining, 2023, 3615-3627.

[27]Hou Z, Liu X, Cen Y, Dong Y, Yang H, Wang C, Tang J. Graphmae: Self-supervised masked graph autoencoders. Proceedings of the 28th ACM SIGKDD Conference on Knowledge Discovery and Data Mining, 2022, 594-604.

[28]Xia J, Zhao C, Hu B, Gao Z, Tan C, Liu Y, Li S, Li SZ. Mole-bert: Rethinking pre-training graph neural networks for molecules. The Eleventh International Conference on Learning Representations, 2023.

[29]Honda S, Shi S, Ueda HR. Smiles transformer: Pre-trained molecular fingerprint for low data drug discovery. arXiv preprint arXiv:191104738, 2019.

[30]Kim H, Lee J, Ahn S, Lee JR. A merged molecular representation learning for molecular properties prediction with a web-based service. Scientific Reports, 2021, 11(1):11028.

[31]Medsker LR, Jain L. Recurrent neural networks. Design and Applications, 2001, 5(64-67):2.

[32]Vaswani A. Attention is all you need. Advances in Neural Information Processing Systems, 2017, 5998-6008.

[33]LaValley MP. Logistic regression. Circulation, 2008, 117(18):2395-2399.

[34]Murtagh F. Multilayer perceptrons for classification and regression. Neurocomputing, 1991, 2(5-6):183-197.

[35]Breiman L. Random forests. Machine learning, 2001, 45:5-32.

[36]Kenton JDM-WC, Toutanova LK. Bert: Pre-training of deep bidirectional transformers for language understanding. arXiv preprint arXiv:181004805, 2019.

[37]Sterling T, Irwin JJ. ZINC 15–ligand discovery for everyone. Journal of chemical information and modeling, 2015, 55(11):2324-2337.

[38]Xu K, Hu W, Leskovec J, Jegelka S. How powerful are graph neural networks? arXiv preprint arXiv:181000826, 2018.

[39]Kipf TN, Welling M. Semi-supervised classification with graph convolutional networks. arXiv preprint arXiv:160902907, 2016.

[40]Hu W, Liu B, Gomes J, Zitnik M, Liang P, Pande V, Leskovec J. Strategies for pre-training graph neural networks. arXiv preprint arXiv:190512265, 2019.

[41]Velickovic P, Fedus W, Hamilton WL, Liò P, Bengio Y, Hjelm RD. Deep graph infomax. ICLR (Poster), 2019, 2(3):4.

[42]You Y, Chen T, Shen Y, Wang Z. Graph contrastive learning automated. International Conference on Machine Learning, 2021. PMLR, 12121-12132.

[43]Hamilton W, Ying Z, Leskovec J. Inductive representation learning on large graphs. Advances in neural information processing systems, 2017, 30.

[44]Liu S, Wang H, Liu W, Lasenby J, Guo H, Tang J. Pre-training molecular graph representation with 3d geometry. arXiv preprint arXiv:211007728, 2021.

[45]Huang J, Rauscher S, Nawrocki G, Ran T, Feig M, de Groot BL, Grubmüller H, MacKerell AD, Jr. CHARMM36m: an improved force field for folded and intrinsically disordered proteins. Nature methods, 2017, 14(1):71-73.

[46]Jo S, Kim T, Iyer VG, Im W. CHARMM-GUI: a web-based graphical user interface for CHARMM. Journal of computational chemistry, 2008, 29(11):1859-1865.

[47]Lee J, Cheng X, Swails JM, Yeom MS, Eastman PK, Lemkul JA, Wei S, Buckner J, Jeong JC, Qi Y *et al*. CHARMM-GUI Input Generator for NAMD, GROMACS, AMBER, OpenMM, and CHARMM/OpenMM Simulations Using the CHARMM36 Additive Force Field. Journal of chemical theory and computation, 2016, 12(1):405-413.

[48]Kim S, Lee J, Jo S, Brooks CL, 3rd, Lee HS, Im W. CHARMM-GUI ligand reader and modeler for CHARMM force field generation of small molecules. Journal of computational chemistry, 2017, 38(21):1879-1886.

[49]Abraham MJ, Murtola T, Schulz R, Páll S, Smith JC, Hess B, Lindahl E. GROMACS: High performance molecular simulations through multi-level parallelism from laptops to supercomputers. SoftwareX, 2015, 1:19-25.

[50]Valdés-Tresanco MS, Valdés-Tresanco ME, Valiente PA, Moreno E. gmx_MMPBSA: A New Tool to Perform End-State Free Energy Calculations with GROMACS. Journal of chemical theory and computation, 2021, 17(10):6281-6291.

[51]Perera TPS, Jovcheva E, Mevellec L, Vialard J, De Lange D, Verhulst T, Paulussen C, Van De Ven K, King P, Freyne E *et al*. Discovery and Pharmacological Characterization of JNJ-42756493 (Erdafitinib), a Functionally Selective Small-Molecule FGFR Family Inhibitor. Molecular cancer therapeutics, 2017, 16(6):1010-1020.

[52]Sootome H, Fujita H, Ito K, Ochiiwa H, Fujioka Y, Ito K, Miura A, Sagara T, Ito S, Ohsawa H *et al*. Futibatinib Is a Novel Irreversible FGFR 1-4 Inhibitor That Shows Selective Antitumor Activity against FGFR-Deregulated Tumors. Cancer research, 2020, 80(22):4986-4997.

[53]Harris PA, Boloor A, Cheung M, Kumar R, Crosby RM, Davis-Ward RG, Epperly AH, Hinkle KW, Hunter III RN, Johnson JH. Discovery of 5-[[4-[(2, 3-dimethyl-2 H-indazol-6-yl) methylamino]-2-pyrimidinyl] amino]-2-methyl-benzenesulfonamide (Pazopanib), a novel and potent vascular endothelial growth factor receptor inhibitor. Journal of medicinal chemistry, 2008, 51(15):4632-4640.

[54]Zhang ZX, Jin WJ, Yang S, Ji CL. BRAF kinase inhibitor exerts anti-tumor activity against breast cancer cells via inhibition of FGFR2. American journal of cancer research, 2016, 6(5):1040-1052.

[55]Li J, An B, Song X, Zhang Q, Chen C, Wei S, Fan R, Li X, Zou Y. Design, synthesis and biological evaluation of novel 2,4-diaryl pyrimidine derivatives as selective EGFR(L858R/T790M) inhibitors. European journal of medicinal chemistry, 2021, 212:113019.

[56]Gao Y, Zhang P, Cui A, Ye DY, Xiang M, Chu Y. Discovery and anti-inflammatory evaluation of benzothiazepinones (BTZs) as novel non-ATP competitive inhibitors of glycogen synthase kinase-3β (GSK-3β). Bioorganic & medicinal chemistry, 2018, 26(20):5479-5493.

[57]Kang C. Infigratinib: First Approval. Drugs, 2021, 81(11):1355-1360.

[58]Wang X, Chen Z, Tong L, Tan S, Zhou W, Peng T, Han K, Ding J, Xie H, Xu Y. Naphthalimides exhibit in vitro antiproliferative and antiangiogenic activities by inhibiting both topoisomerase II (topo II) and receptor tyrosine kinases (RTKs). European journal of medicinal chemistry, 2013, 65:477-486.

[59]Kooijman JJ, van Riel WE, Dylus J, Prinsen MBW, Grobben Y, de Bitter TJJ, van Doornmalen AM, Melis J, Uitdehaag JCM, Narumi Y *et al*. Comparative kinase and cancer cell panel profiling of kinase inhibitors approved for clinical use from 2018 to 2020. Frontiers in oncology, 2022, 12:953013.

[60]Menichincheri M, Ardini E, Magnaghi P, Avanzi N, Banfi P, Bossi R, Buffa L, Canevari G, Ceriani L, Colombo M. Discovery of entrectinib: a new 3-aminoindazole as a potent anaplastic lymphoma kinase (ALK), c-ros oncogene 1 kinase (ROS1), and pan-tropomyosin receptor kinases (Pan-TRKs) inhibitor. Journal of medicinal chemistry, 2016, 59(7):3392-3408.

[61]Wu L, Zhang C, He C, Qian D, Lu L, Sun Y, Xu M, Zhuo J, Liu PCC, Klabe R *et al*. Discovery of Pemigatinib: A Potent and Selective Fibroblast Growth Factor Receptor (FGFR) Inhibitor. Journal of medicinal chemistry, 2021, 64(15):10666-10679.

[62]Matsui J, Yamamoto Y, Funahashi Y, Tsuruoka A, Watanabe T, Wakabayashi T, Uenaka T, Asada M. E7080, a novel inhibitor that targets multiple kinases, has potent antitumor activities against stem cell factor producing human small cell lung cancer H146, based on angiogenesis inhibition. International journal of cancer, 2008, 122(3):664-671.

[63]Wolfe A, O'Clair B, Groppi VE, McEwen DP. Pharmacologic characterization of a kinetic in vitro human co-culture angiogenesis model using clinically relevant compounds. Journal of biomolecular screening, 2013, 18(10):1234-1245.

[64]Rolf MG, Curwen JO, Veldman-Jones M, Eberlein C, Wang J, Harmer A, Hellawell CJ, Braddock M. In vitro pharmacological profiling of R406 identifies molecular targets underlying the clinical effects of fostamatinib. Pharmacology research & perspectives, 2015, 3(5):e00175.

[65]Liu Y, Jin S, Peng X, Lu D, Zeng L, Sun Y, Ai J, Geng M, Hu Y. Pyridazinone derivatives displaying highly potent and selective inhibitory activities against c-Met tyrosine kinase. European journal of medicinal chemistry, 2016, 108:322-333.
